# Microsecond-Scale Transient Thermal Sensing Enabled by Flexible Mo_1−x_W_x_S_2_ Alloys

**DOI:** 10.34133/research.0452

**Published:** 2024-08-21

**Authors:** Weiwei Li, Lingyan Kong, Manzhang Xu, Jiuwei Gao, Lei Luo, Yingzhe Li, Kexin Wang, Yilin Zhou, Lei Li, Yuan Wei, Xiaoshan Zhang, Ruoqing Zhao, Mengdi Chen, Yuting Yan, Xiaoguang Luo, Zhaohe Dai, Lu Zheng, Xuewen Wang, Wei Huang

**Affiliations:** ^1^ Frontiers Science Center for Flexible Electronics (FSCFE) & Shaanxi Institute of Flexible Electronics (SIFE), Northwestern Polytechnical University (NPU), Xi’an 710072, China.; ^2^ Shaanxi Key Laboratory of Flexible Electronics (KLoFE), Northwestern Polytechnical University (NPU), Xi’an 710072, China.; ^3^ MIIT Key Laboratory of Flexible Electronics (KLoFE), Northwestern Polytechnical University (NPU), Xi’an, 710072, China.; ^4^ State Key Laboratory of Organic Electronics and Information Displays, Institute of Advanced Materials (IAM), Nanjing University of Posts & Telecommunications, Nanjing, 210023, China.; ^5^ Key Laboratory of Flexible Electronics (KLoFE) and Institute of Advanced Materials (IAM), Nanjing Tech University (NanjingTech), Nanjing, 211800, China.; ^6^ Department of Mechanics and Engineering Science, College of Engineering, Peking University, Beijing 100871, China.

## Abstract

Real-time thermal sensing through flexible temperature sensors in extreme environments is critically essential for precisely monitoring chemical reactions, propellant combustions, and metallurgy processes. However, despite their low response speed, most existing thermal sensors and related sensing materials will degrade or even lose their sensing performances at either high or low temperatures. Achieving a microsecond response time over an ultrawide temperature range remains challenging. Here, we design a flexible temperature sensor that employs ultrathin and consecutive Mo_1−*x*_W*_x_*S_2_ alloy films constructed via inkjet printing and a thermal annealing strategy. The sensing elements exhibit a broad work range (20 to 823 K on polyimide and 1,073 K on flexible mica) and a record-low response time (about 30 μs). These properties enable the sensors to detect instantaneous temperature variations induced by contact with liquid nitrogen, water droplets, and flames. Furthermore, a thermal sensing array offers the spatial mapping of arbitrary shapes, heat conduction, and cold traces even under bending deformation. This approach paves the way for designing unique sensitive materials and flexible sensors for transient sensing under harsh conditions.

## Introduction

Real-time sensing over an ultrabroad temperature range is crucial due to its potential applications in extreme environments, such as aerospace, oceans, and chemical industrial systems [1–3]. Especially, flexible thermal sensors provide a novel and unique approach to substantially improving signal quality by conforming to rigid and complex surfaces. For practical applications, it is essential for such devices to achieve real-time and accurate thermal sensing while maintaining regular operation without deterioration in sensing performances under extreme temperature conditions [4–8].

Rapid advances in material science enable the emergence of unique sensitive materials with high mechanical flexibility for high-temperature applications, including semiconductors [7,9–12], carbon nanomaterials [13–15], transition metal carbides [16,17], and metals[18–21]. Organic semiconductors are flexible but may degrade or even lose their sensitive properties at high temperatures [22–25]. It is generally difficult for ceramics and transition metal oxides to achieve flexibility for temperature sensors [26–28], even composited with organic materials [29–32]. Metal oxides and graphene-based thermal sensors could operate at temperatures higher than 1,500 K [4,33]. However, limited by the sensing mechanism, these sensors typically have a large response time (even longer than 1 s) [6,34]. In contrast, metallic materials exhibit a fast response to temperature change on a millisecond scale and operate at high temperatures over 1,000 K [7,35]. However, the sensitivity to temperature is much lower than semiconductors and carbon nanomaterials [36,37]. Recently, transition metal dichalcogenides (TMDs) with atomic layer structure have been proven to perform fast response and high-temperature coefficient of resistance (TCR) [12,38–40], which is promising for real-time thermal sensing. However, their performance over a broad temperature regime from extremely low to high is yield exploring.

In this work, we report the design and realization of Mo_1−*x*_W*_x_*S_2_ alloy films (0 ≤ *x* ≤1) and the integration with interdigital electrodes to realize flexible temperature sensing arrays adaptable for extreme temperature conditions. The sensors can simultaneously achieve high sensitivity (i.e., TCR), fast response, low detection limit, and excellent robustness over an ultrawide temperature range. Superior sensing performances are successfully retained even under harsh environments, such as immersion in liquid nitrogen (N_2_), exposure to fire flame, and rapid thermal shock. Moreover, due to the intrinsically fast response to temperature, the Mo_1−*x*_W*_x_*S_2_ alloy-based temperature sensors enable the instantaneous detection of various temperature signals induced by liquid N_2_, water droplets, and flames.

## Results

### Architecture of the flexible sensor and sensing array

**Fig. 1.**
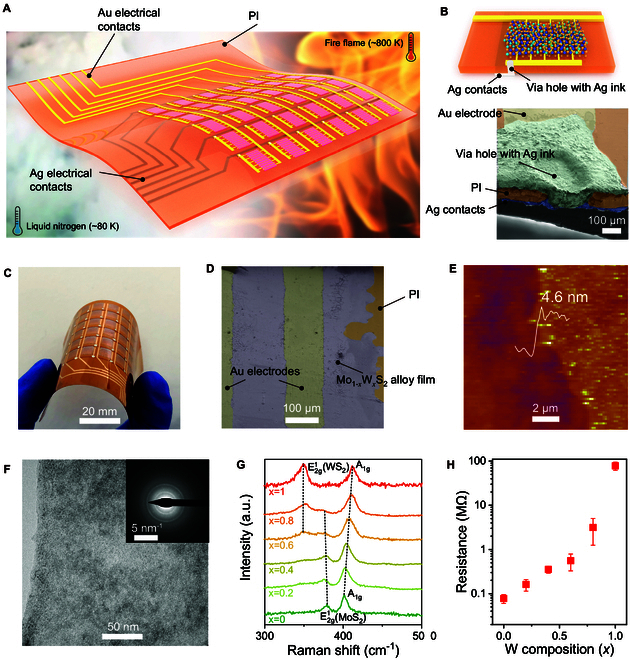
Design and architecture of the Mo_1−*x*_W*_x_*S_2_ alloy-based sensing array. (A) Three-dimensional diagram of a temperature sensing array. (B) Diagram of a single sensing element and the corresponding cross-sectional scanning electron microscopy (SEM) image of the via holes with Ag inks. (C) Photographic image demonstrating the fabricated sensor array under bending deformation. (D) SEM image of the Mo_1−*x*_W*_x_*S_2_ alloy film on PI and Au interdigital electrodes. (E) AFM image of the printed alloy film with an ink concentration of 2 mg ml^−1^ in a single printing pass. (F) HR-TEM image of the synthesized Mo_0.6_W_0.4_S_2_ alloy. The inset shows the corresponding SAED pattern. (G) Raman spectra of the Mo_1−*x*_W*_x_*S_2_ alloy thin films on PI substrates for *x* = 1, 0.8, 0.6, 0.4, 0.2, and 0. (H) Resistance of Mo_1−*x*_W*_x_*S_2_ alloy thin films against W composition, *x*, with steps of 0.2.

Figure 1A shows a schematic architecture of the proposed flexible sensing array, consisting of 6 × 6 sensing elements comprising Mo_1−*x*_W*_x_*S_2_ films, gold (Au) interdigital electrodes, and electrical contacts for temperature sensing, data acquiring, and signal transmission. All the components are fabricated on a 100-μm-thick polyimide (PI) film to ensure high flexibility for intimate integration with curved surfaces. An electrical contact array with a crossover structure is designed on both sides of the PI film to minimize the lead numbers (from 72 leads for the planar structure to 12 leads). Laser drilling is used to create holes at the end of one interdigital branch with a dimension of about 0.4 mm. We then fill the holes with silver (Ag) ink to connect the Au electrodes with the Ag contacts on the reverse side of PI (Fig. 1B). Mo_1−*x*_W*_x_*S_2_ alloy films with atomically layered consecutive structures are constructed by a combination of inkjet printing and thermal annealing strategy, enabling the high-efficiency fabrication of flexible temperature sensors and sensor array over a large area (Fig. 1C). We provide a schematic illustration of the overall synthesis scheme for the Mo_1−*x*_W*_x_*S_2_ alloys in Fig. S1A. Optimal precursor inks comprising ammonium thiomolybdate [(NH_4_)_2_MoS_4_] and ammonium tetrathiotungstate [(NH_4_)_2_WS_4_] with different Mo to W molar ratios were prepared (Fig. S1B to D and Materials and Methods). Prior to printing, we evaluated the particle sizes of the precursor inks using dynamic light scattering. The results show a narrow particle size distribution in the range of 70 to 120 nm for all the inks (Fig. S2). In addition, an apparent Tyndall scattering effect in the precursor inks is observed (Fig. S2), indicating that the precursors in the solvent mixture are in the form of nanoparticle colloids. The narrow size distribution and small particle size are beneficial for inkjet printing without nozzles blocking. The inks were then deposited on Au interdigital electrodes, which were initially treated by an ultraviolet/ozone (UV/O_3_) for 3 min to improve the wettability and enhance the adhesion with precursor inks by forming hydrophilic groups (i.e., -OH and -COOH) on the surfaces.

Morphological characterizations of the deposited precursors using optical microscopy revealed a uniform coverage on the PI substrate and Au electrodes (Fig. 1D and Fig. S3). Following the inkjet printing, the printed precursors were thermally annealed in a gas mixture of argon (Ar) and hydrogen (H_2_) at ambient pressure. The PI substrates with patterned precursors were then heated to 400 °C and preserved for 20 min, resulting in the thermal decomposition and conversion of the patterned precursors to Mo_1−*x*_W*_x_*S_2_ alloy films. The technical and operational details of the Au electrode scribing, hole drilling, inkjet printing, thermal annealing, and screen printing of Ag electrical connections were discussed in Materials and Methods and Fig. S4. The fabricated sensing array exhibits excellent mechanical flexibility (Fig. 1C), demonstrating potential in application with nonplanar surfaces.

### Controllable synthesis and characterizations of Mo_1−*x*_W*_x_*S_2_ alloys

We first investigate the microstructural characteristics of the Mo_1−*x*_W*_x_*S_2_ alloys. Figure S5A shows a photograph of the printed Mo_1−*x*_W*_x_*S_2_ alloys on a PI substrate with different W composition, *x*, at intervals of 0.2, demonstrating the scalability of our proposed strategy. The physical features of the alloy films, such as layer thickness and pattern sizes, can be feasibly regulated by tailoring the ink concentration and printing parameters, which is the premier advantage in the inkjet printing process [41]. To determine the thickness of the synthesized alloy films, we examine the height profiles by scanning the printed lines with a profiler. A broad tuning range for thickness (Fig. S5B), from 5.1 to 46 nm, was achieved by printing the precursor ink for 1 to 5 printing passes with ink concentrations of 2, 5, and 10 mg ml^−1^. The as-synthesized MoS_2_ thin film with a thickness of about 5.1 nm corresponds to a 7-layer structure. Representative atomic force microscopic (AFM) images (Fig. 1E and Fig. S5C) show thicknesses of 4.6, 7.1, and 12.6 nm for the precursor inks in a single printing pass with ink concentrations of 2, 5, and 10 mg ml^−1^, respectively, which are comparable with the values obtained from the profiler (i.e., 5.1, 7.6, and 13.2 nm). In addition, the printing parameters significantly influence the feature sizes of the printed precursors. For example, the width of the printed lines with a single printing pass shrank from 180 to 49 μm with increased droplet spacing (Fig. S5D and E), indicating the controllability of feature sizes. In addition, dot arrays with a diameter of about 40 μm are also obtained on the substrate (Fig. S5F), demonstrating the relatively high resolution of inkjet printing. Note that the printed line width can be further reduced using a cartridge with a smaller droplet volume. It should also be noted that highly customizable patterns can be directly deposited on the substrates, such as coils, circles, and array structures (Fig. S5G and H).

High-resolution transmission electron microscopy (HR-TEM) was used to examine the crystalline structure of the synthesized films. Figure 1F shows the HR-TEM images of the typical Mo_1−*x*_W*_x_*S_2_ alloy with *x* = 0.4. The synthesized Mo_0.6_W_0.4_S_2_ alloy film was continuous over the selected area and had a polycrystalline structure, which was observed from the selected-area electron diffraction (SAED) pattern (inset in Fig. 1F). We further performed the elemental analysis of the Mo_0.6_W_0.4_S_2_ alloy using energy-dispersive x-ray spectroscopy (EDXS) equipped in HR-TEM. The film consisted of homogeneously distributed Mo, W, and S elements throughout the whole region, as observed in the elemental mapping image (Fig. S6). The calculated W composition, *x*, from the EDXS mapping, is about 0.35, which is close to the value of the precursors (*x* = 0.4). Figure 1G displays the Raman spectra of the Mo_1−*x*_W*_x_*S_2_ alloy at *x* = 0 to 1 in steps of 0.2. For MoS_2_ and WS_2_ thin films, 2 vibration modes of in-plane ($E_{2g}^{1}$) and out-of-plane (*A*_1g_) are observed, with the corresponding peaks located at 379 and 402 cm^−1^ and 349 and 412 cm^−1^, respectively. For the Mo_1−*x*_W*_x_*S_2_ alloys, as the W composition increases, the frequency of *A*_1g_ mode shifts to a higher frequency, while the frequency of $E_{2g}^{1}$ mode for MoS_2_ remained relatively constant. Meanwhile, the peak intensity at 379 cm^−1^ was gradually reduced, suggesting a reduction in the content of MoS_2_ in the Mo_1−*x*_W*_x_*S_2_ alloy. It is also observed that a new peak related to the $E_{2g}^{1}$ mode of WS_2_ appeared at about 349 cm^−1^ when the W composition was increased to 0.6. The intensity of this peak increased with the increase in W composition. Overall, there is a 1-mode and 2-mode behavior for *A*_1g_ and $E_{2g}^{1}$ modes (Fig. S7A to C), respectively, demonstrating a composition dependence in the Raman spectra of the Mo_1−*x*_W*_x_*S_2_ alloy [42]. The trend of the Raman spectra of the Mo_1−*x*_W*_x_*S_2_ alloy with different W compositions are consistent with the synthesized Mo_1−*x*_W*_x_*S_2_ alloys using other methods, such as atomic layer deposition [43] and chemical vapor deposition (CVD) [44–46].

Apart from the Raman spectra, the photoluminescence (PL) spectra of the resultant alloy films with a full W composition from *x* = 0 to *x* = 1 at steps of 0.2 were taken to investigate the PL property. As a comparison, the PL spectra of monolayer, bilayer MoS_2_, and WS_2_ are also taken from the grown single-crystalline structures in our laboratory. As expected, the PL intensity of the alloy films is extremely weak compared to the single-crystalline monolayer and bilayer structures (Fig. S7D to F). It is consistent with the reported results [47], indicating bulk cases with indirect bandgaps. We also conducted x-ray photoelectron spectroscopy (XPS) to verify the atomic composition of the synthesized films. Figure S7G and H shows the XPS spectra of the films with various W compositions. The WS_2_ (*x* = 1) had binding energies related to W4f at about 35.5 and 32.4 eV. The peak intensity at 35.5 eV gradually decreased with the reduction in W composition. In contrast, the peak for Mo3d located at about 232.5 and 229.3 eV appeared when the W composition was reduced to 0.8. The peak intensity of Mo3d roughly increased with a decrease in *x* value, suggesting the increased Mo elements in the Mo_1−*x*_W*_x_*S_2_ alloys. X-ray diffraction patterns were obtained to characterize the crystalline structures of the alloys. For the MoS_2_ and a typical alloy (Mo_0.4_W_0.6_S_2_), the crystalline phase signals are from MoS_2_ and WS_2_ (Fig. S7I), confirming the formation of Mo_1−*x*_W*_x_*S_2_ alloys without impurity.

The composition of the alloys plays a crucial role in controlling the electrical performance of the synthesized films. By tuning the W composition, *x*, a gradual and continuous variation in the resistance was achieved (Fig. 1H), wherein the resistance increased from 0.16 MΩ at *x* = 0.2 to 78.3 MΩ at *x* = 1 (with 5 printing passes). This tunability in resistance can be attributed to the doping of W atoms into MoS_2_ [46]. It is reported that the charge transport of semiconductors highly depends on the dynamics of carriers. We evaluated the carrier concentration and carrier mobility of the alloy films by Hall measurement (Fig. S8A and B). As expected, the carrier concentration and mobility of MoS_2_ are relatively larger than that of WS_2_ due to the wider bandgap (Fig. S8C) [48,49]. Moreover, substituting W atoms for Mo atoms in Mo_1−*x*_W*_x_*S_2_ (0 < *x* < 1) has mediated carrier concentrations. Therefore, for a given temperature, the electrical conductivity of the W-doped MoS_2_ gradually decreases with an increase in the W composition. Figure S5F shows the correlation between the resistance of the printed alloys and the number of printing passes. The resistance of MoS_2_ films decreased linearly from about 0.25 to 0.07 MΩ as the printing pass increased from 1 to 5, which can be ascribed to the increased thickness of the printed layer. These observations confirm the successful synthesis of MoS_2_, WS_2_, and alloyed Mo_1−*x*_W*_x_*S_2_ with controllable compositions and electrical performance using the proposed printing-and-annealing strategy.

### Static and dynamic sensing performance

We then investigated the electrical response of the printed-and-annealed Mo_1−*x*_W*_x_*S_2_ alloys on the PI substrate by varying the temperature from 20 to 823 K. Note that the ultrawide temperature range is separated by 303 K due to limitations of the temperature source used for the measurement (see details in Materials and Methods). The tests in 20 to 80 K, 80 to 303 K, and 303 to 823 K were indicated by ultralow-temperature, low-temperature, and high-temperature tests, respectively. Figure S9 shows photographs of the experimental setups for evaluating the response of the sensor under different temperatures. In the ultralow-temperature measurement, *I*–*V* curves at different temperatures are recorded using a semiconductor analyzing platform with a probe station. For the low-temperature test, a precious temperature controller with a resolution of 0.01 K was used to apply temperature gradient onto the sensors by filling liquid N_2_ into a chamber surrounding the sensor. For the high-temperature test, a tube furnace equipped with a homemade sample holder provided gradient temperatures to the sensors. The fractional changes in resistance were recorded using a digital multimeter. It should be noted that the temperature budget for the test is 823 K, which is the maximum temperature for PI films. Higher temperatures will result in a structure change or even damage to the PI films (Fig. S10).

**Fig. 2.**
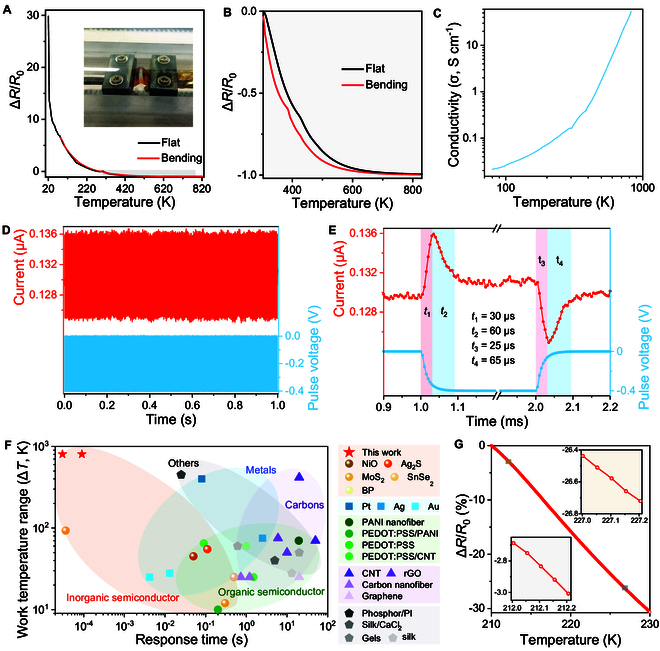
Temperature sensing properties of Mo_1−*x*_W*_x_*S_2_ alloy-based temperature sensors. (A) Relative resistance variation of the sensor upon increasing the temperatures from 20 to 823 K at flat and under bending conditions. The inset is a photograph of the sensor under bending. (B) Resistance change in the range of 303 to 823 K. (C) Log-log plot of electrical conductivity versus temperature for the alloy sensor. (D) Resistance change of the sensor and the pulsed voltage versus time over 1,000 cycles. The resistance variation is induced by the increased temperature, which is derived from the heater. (E) Extracted response time of the sensor. The activation time for heating and cooling are *t*_1_ = 30 μs and *t*_2_ = 25 μs, respectively. Stabilized time for heating and cooling are *t*_h_ = *t*_1_ + *t*_2_ = 90 μs and *t*_c_ = *t*_3_ + *t*_4_ = 90 μs, respectively. (F) Comparison in terms of response time and work temperature range among the proposed sensor and the temperature sensors in the literature. (G) Relative resistance change for the sensor as a function of temperature from 210 to 230 K. The insets show the signals at 212 to 212.2 K and 227 to 227.2 at steps of 0.05 K, exhibiting a high linearity.

Figure 2A and B displays the relative resistance change (Δ*R*/*R*_0_) of the Mo_0.2_W_0.8_S_2_ alloy sensor with temperature. According to our measurement setup, we define the resistance at 303 K as the initial value (i.e., *R*_0_) for calculating resistance change and the following temperature coefficient [50]. Thus, positive values of Δ*R*/*R*_0_ at the low-temperature range (i.e., 20 to 303 K) were obtained, while the values of Δ*R*/*R*_0_ were negative from 303 to 823 K. Overall, the electrical resistance decreased with the increase in temperature, demonstrating a typical semiconductor behavior [50]. In specific, there is a gradual increase in the electrical conductivity from 0.02 to 0.16 S cm^−1^ when the temperature increased from 80 to 300 K (Fig. 2C). As the temperature increased further from 303 to 823 K, a rapid increase in the electrical conductivity up to 53 S cm^−1^ was observed.

We further calculated the sensitivity (*S*) of the resistive-type alloy-based temperature sensor, which is defined as *S* = (Δ*R*/*R*_0_)/Δ*T*, where *R* is the measured resistance at temperature *T*. The data indicated reasonable sensitivities over an ultrawide temperature range. Specifically, when the temperature varied from 20 to 250 K, the averaged sensitivity decreased from 5.02 to 0.82 % K^−1^ (Fig. S11A). In the high-temperature regime (>303 K), the Mo_0.2_W_0.8_S_2_ alloy sensor exhibited a sensitivity ranging from −0.88 to −0.63 % K^−1^. Note that the Mo_1−*x*_W*_x_*S_2_ alloy temperature sensors with different W compositions from *x* = 0 to *x* = 0.6 exhibit similar resistance change over the entire temperature range from 80 to 823 K (Fig. S11B to D), despite the slight variations in the calculated TCRs (Fig. S11E to I). In addition, the Mo_1−*x*_W*_x_*S_2_ alloy sensors are mechanically flexible, wherein the sensing performance can be retained under bending deformation. The calculated sensitivities coincided under flat and bending conditions at various temperatures (Fig.S11A), indicating that the Mo_1−*x*_W*_x_*S_2_ alloy-based sensors were suitable for flexible sensing applications.

We conducted further investigations on the flexible Mo_1−*x*_W*_x_*S_2_ alloy sensor by depositing an Ag line as a heater (Fig. S12) to determine its response time. Note that a thin PI layer of about 300 nm is introduced as an isolation layer to avoid the electrical short between the heater and the Au electrodes. We applied alternative voltage pulses with an amplitude of −0.4 V and a pulse duration of 1 ms to the heater. The generated heat during the pulsed periods was then spread to the alloy layer through the isolation layer, resulting in an increase in the current of the sensor. Upon removal of the voltages, the temperature of the heater and the sensor started decreasing, indicating a recovery in the current. Using a semiconductor analyzer, we can record the pulsed voltages and the electrical response of the alloy sensor simultaneously. As shown in Fig. 2D, the resistance of the sensor decreased on the activation of the heater after being pulsed with voltages. A stable and reversible response to the pulsed heating was achieved for 1,000 cycles. It is observed that there was an immediate response for the alloy sensor to the pulsed voltages. The time to reach the maximum temperature was about 30 μs, while it took 90 μs to stabilize the response (Fig. 2E), which was consistent with the thermal time. Upon removing the pulsed voltages, the currents in the sensor recovered to their initial values within 90 μs. The transient response to temperature could be attributed to the thin-film structure and the fast charge carrier transport in atomically layered structure [38]. Note that there is a rapid variation in the current for the sensor upon pulsed actuation and removal. It was ascribed to the transient surges from the instrument, which can be mitigated by the integration with a surge protector [51,52].

We then compare the sensing performance of our Mo_1−*x*_W*_x_*S_2_ alloy-based temperature sensors with the state-of-the-art temperature sensors in the literature in terms of work temperature range and response time. A thorough exploration of various thermal-sensitive materials, including semiconductors [TMDs [39], Ag_2_S [50], SnSe_2_ [9], nickel (II) oxide [26], black phosphorus (BP) [53], poly(3,4-ethylenedioxythiophene):poly(styrene sulfonate) (PEDOT:PSS) [54,55], and polyaniline (PANI) [23,56]], metals (Pt [57], Au [58], and Ag [59–61]), carbon nanomaterials [carbon nanofibers [62], carbon nanotubes (CNTs) [6,63], graphene [14,64,65], and graphene oxide [66]], and several other functional materials (phosphor/PI [1], silk/CaCl_2_ [67], and gels [68,69]) (Table S1) have been conducted for the comparison. Figure 2F demonstrates the competitiveness of the proposed Mo_1−*x*_W*_x_*S_2_ alloy-based temperature sensors. It highlights their promising prospects for fast detection of temperature variations across an ultrabroad sensing range from a low temperature of liquid N_2_ to a high temperature of flame burning. It should be noted that the proposed approach is general for depositing Mo_1−*x*_W*_x_*S_2_ precursors on the substrate to realize optoelectronic devices, which is expected to further extend the work temperature range of the sensors. We confirm this by fabricating a flexible temperature sensor by depositing the alloy films on thermal-resistant mica substrate, exhibiting a higher operating temperature of up to 1,073 K (Fig. S13).

In addition to the ultrawide response range and fast response time, the Mo_1−*x*_W*_x_*S_2_ alloy-based temperature sensors could discriminate subtle temperature variation of 0.05 K with excellent linearity in the temperature range of 212 to 212.2 K and 227 to 227.2 K (Fig. 2G). Moreover, a temperature change of about 0.02 K was also accurately recorded at the low temperature of 100 and 220 K, as well as at a high temperature of 700 K (Fig. S14). It should be noted that the linearity of the curves at lower temperatures is slightly larger than that at higher temperatures, which can be attributed to the decreased sensitivity of the sensor with the rise of temperature. Nevertheless, these results suggested that the Mo_1−*x*_W*_x_*S_2_ alloy-based temperature sensors possessed the ability to detect tiny temperature changes (0.02 and 0.05 K) over a broader temperature range (about 100 to 700 K), which was superior to that of most temperature sensors in the literature (Table S1).

The fast response, wide working range, and satisfactory resolution of our Mo_1−*x*_W*_x_*S_2_ alloy-based temperature sensor enable it to detect instantaneous temperature variations. For instance, when liquid N_2_ was poured onto the surface of the sensor, we observed a dramatic increase in resistance upon contact with the liquid N_2_ droplets (Fig. S15A and Movie S1). Subsequently, the resistance decreased dramatically, which was attributed to the rapid vaporization of liquid N_2_. Additionally, the sensor demonstrated sensitivity to water with different temperatures. In this experiment, hot water droplets were dripped onto the sensor, and its resistance exhibited an instant decrease upon contact with the droplets (Fig. S15C and D). Interestingly, the sensor also responded to room temperature water (Fig. S15E and F), wherein a slight decrease in resistance of about 1.2% was observed for water droplets at 293 K (the same temperature as the sensor). It suggests that our sensor was capable of capturing tiny temperature differences in water droplets. These results demonstrate the ability of the sensor to dynamically and accurately measure the surface temperature of the sensor in a fast and reliable manner.

The fast response was also observed at a high-temperature range by moving a fire flame to the sensor. The corresponding resistance change was displayed in Fig. S15B and Movie S12. The sensor exhibited swift responses when the fire was at “ON” and “OFF” states, further confirming the fast response time of the Mo_1−*x*_W*_x_*S_2_ alloy-based sensor. The detection of such instantaneous temperature change takes a longer time using commercially available thermocouples (TCs) (Fig. S16A and B). More importantly, the Mo_1−*x*_W*_x_*S_2_ alloy-based sensor was capable of distinguishing the instantaneous fluctuation of a flame during burning (Fig. S16C and D), which was verified by the oscillations in the electrical response. This further suggested that our sensor responded swiftly and sensitively to dynamic vibrations in temperature, which was not observed in the output of a commercial TC (Movie S3).

**Fig. 3.**
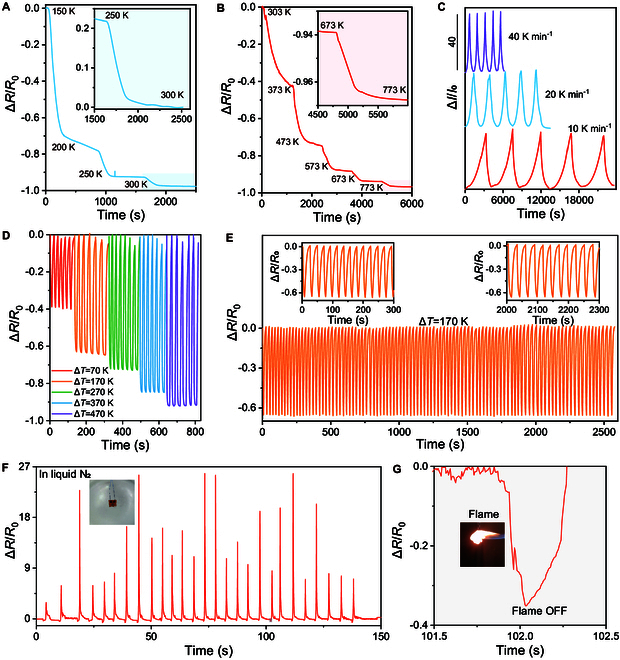
Dynamic sensing behavior and signal stability of the Mo_1−*x*_W*_x_*S_2_ alloy-based temperature sensors. Relative resistance variation during the programmable heating and cooling (A) from 150 to 300 K, and (B) from 303 to 773 K with a holding time of 5 min at each step. Insets in (A) and (B) show the enlarged curves from 250 to 300 K, and 673 to 773 K, respectively. (C) Relative resistance change of the sensor under a temperature variation of 470 K at different heating frequencies of 10, 20, and 40 K min^−1^, showing consistent and repeatable sensing response. (D) Relative changes in resistance as a function of a dynamic sweep at various temperature gradients from 70 to 470 K at steps of 100 K. (E) Durability test for the sensor under 100 cyclic heating and cooling with a temperature gradient of 170 K at a frequency of about 0.05 Hz. Insets show the relative resistance changes over the initial 300 s and the last 300 s for the cyclic heating. (F) Cyclic resistance change of the sensor under repeatable heating by a fire flame followed by immersion in liquid N_2_. Insets show the photographs of the sensor under burning and in liquid N_2_. (G) The enlarged curve for the electrical response in (F) from 101.5 to 102.5 s shows the resistance change when the sensor is subjected to flame, the removal of flame, and immersion in liquid N_2_.

We further applied a programmable increment in temperature to the sensor at both the low- and high-temperature tests with a hold time of 5 min at each temperature. As shown in Fig. 3A and B, the sensor responded immediately after the temperature varied. The resistance remained nearly constant when the temperature reached the set value regardless of the temperature range. It should be noted that the resistance still varied at some temperatures, such as 200 and 373 K, which could be attributed to the low-precision heating source and the uncapped alloy layer. The tube furnace used for the heating was relatively imprecise in controlling the temperature at the initial stage. The temperature kept increasing even after the set value was settled. Thus, increasing the hold time is feasible for stabilizing the temperature. On the other hand, air and moisture have been absorbed on the surface of the pristine alloy layer, which had a significant influence on the stable response of temperature sensors [38]. We also evaluated the response of the sensor by applying increased heating frequencies from 10 to 40 K min^−1^. As shown in Fig. 3C, the electrical response was independent of heating frequencies, wherein the normalized current maintained the shapes and peaks regardless of the subjected heating frequencies, suggesting that the proposed temperature sensor is highly reproducible under different operating conditions.

The dynamic behavior of the alloy sensor was investigated by subjecting thermal shock with different temperature gradients of 70, 170, 270, 370, and 470 K. At each temperature level, repeatable changes in resistance over multiple cycles of heating and cooling were observed (Fig. 3D). Moreover, there had incremental increases in resistance change as a function of temperature gradients with stepwise heating from 70 to 470 K. After cooling to room temperature, the resistance returned to the initial values, demonstrating a reliable and rapid response in detecting various temperature gradients. Moreover, the long-term stability and durability were examined to investigate the ability to retain sensing performance under repeated heating and cooling tests. As expected, high reproducibility of electrical response was recorded over 100 heating/cooling cycles with an identical temperature gradient of 170 K (Fig. 3E). We also investigated the resistance change of the sensor to small temperature changes. As shown in Fig. S17, there are identical signals when temperature gradients of about 2, 4, and 8 K were applied to the sensor, further confirming the high repeatability of the sensor. Specifically, the signal of the sensor remained constant throughout the entire cyclic test without obvious fluctuation in the heating and cooling curves, demonstrating a reliable and durable output. It is crucial for the practical applications of flexible temperature sensors. The sensors were also subjected to a rigorous test to further evaluate their stability. The Mo_1−*x*_W*_x_*S_2_ alloy-based sensor was exposed to a combustion environment by applying a flame with a temperature of about 600 K, followed by being subjected to cryogenic temperature via immersion in liquid N_2_ (Fig. 3F and G). Upon exposure to flame, the temperature of the sensor increased immediately, resulting in a rapid decrease in resistance (Fig. 3G). Note that the fluctuation of the resistance variation was attributed to the unstable flame during burning, which verified the high sensitivity of the sensor. Before cooling to room temperature, the sensor was dipped into liquid N_2_. Positive values of resistance variation were instantaneously observed when the sensor came into contact with liquid N_2_ (Fig. 3F). After removing the external stimuli, the sensors recovered to the initial electrical property. The results revealed that the sensor responded precisely to the alternative heating and cooling applied.

Apart from the aforementioned results, it is reported that the in-plane thermal expansion coefficient (TEC) of 2D transition metal dichalcogenide films with thicknesses of few nanometers are from 6 to 10 ppm K^−1^ [70,71], which are lower than that of the PI films (i.e., typically larger than 30 ppm K^−1^) [72,73]. The large TEC mismatch may lead to thermal stress (i.e., tensile or compressive stress) or stress concentration within the inorganic materials, resulting in delamination or peeling off from PI substrates [74]. As a result, the signal output of the devices may be unstable at elevated temperatures. However, we found that the resistance changes of our sensors are highly stable at a broad temperature range (i.e., 550 K) or dynamic tests for multiple heating/cooling cycles. Additionally, the resistance remains relatively stable under a high temperature of 800 K, even for more than 8 h (Fig. S18). These results indicate that there is no delamination between the alloy layers and PI films nor damage to the sensors. The underlying mechanisms could be derived from the strong contact and the high conformability of the alloy layer to the PI films. In our experiments, UV/O_3_ treatment was applied to the PI films before depositing the precursor inks, leading to solid bonding of precursor molecules with the hydrophilic functional groups on the surface of PI films. More importantly, the printed precursors are decomposed and in situ grown on PI films, providing ultrahigh adhesion between the annealed alloys and the PI films even after the heating experiments (Fig. S19). We also approximately analyze the thermal stress *σ_T_* and its mechanical effect caused during the heating or cooling process (Fig. S20). We may estimate the magnitude of thermal stress within the alloy layer via *σ_T_*~Δ*α*Δ*T* ⋅ *E*, where Δ*α* is the mismatched coefficient of thermal expansion between the alloy materials in the system, Δ*T* ~ 500 K is the range of the operating temperature (relative to the ambient reference), and *E* ~ 270 GPa is Young’s modulus of the PI film. Based on the Griffith condition [75], the driving force for the interfacial delamination to occur is given by the energy release rate $\varsigma \sim \frac{1-\nu^{2}}{2E}\sigma_{T}^{2}t$ , where *ν* and *t* are Poisson’s ratio and thickness of the Mo_1−*x*_W*_x_*S_2_ alloy, respectively. The largest driving force is found to be ~0.14 J/m^2^ in the thickest Mo_1−*x*_W*_x_*S_2_ films (~46 nm), which is much smaller than the delamination energy of typical 2-dimensional (2D) material–polymer interface (~1 J/ m^2^ measured by standard double cantilever beam tests [76]). It explains the mechanical advantage of our sub-100-nm-thick sensors, particularly their mechanical robustness upon heating and cooling. These results indicated superior stability and resistance to both low-temperature and high-temperature environments, enabling our sensors to be potentially used in several critical scenarios, such as in outer space.

We also investigated the stability of the sensor in other environments. The storage stability of the sensor was first evaluated by investigating the resistance change after being stored at ambient conditions with an environment temperature of about 25°C and a relative humidity of about 60%. The resistance increased by about 27% after 6 months (Fig. S21A). The sensors were also soaked in the solutions with different pH values from 2 to 14. As shown in Fig. S21B, there was less than a 20% increase in resistance when the sensor was soaked in acid solutions for 30 min, indicating that the sensor could withstand strong acid corrosion. However, the resistance of the sensor increased by more than 50% and 3 times in alkaline solutions (pH values of 9 and 14) for 10 and 30 min, respectively. Encapsulation is required for the sensor to be used in alkaline solutions. We also evaluated the proper functions of the sensor in various solutions, including deionized water, drinking water, acid solution (pH 4), and ethanol. As shown in Fig. S22, there was an increase in resistance when the sensor was immersed in these solutions before it reached a steady value. Then, we added few droplets of hot water into the solution to change the temperature. As expected, the sensor responded immediately after adding the hot water droplets, indicating the proper working of the sensor in both the atmosphere and various solutions.

### Spatial mapping

A temperature sensing array of 6 × 6 sensing elements over an area of 5 cm × 5 cm, with each sensing element being a square sensor with a side length of 3.5 mm, is realized to demonstrate the scalability of the proposed strategy and the suitability of the Mo_1−*x*_W*_x_*S_2_ alloy-based sensor for practical applications. The fabricated sensor array exhibited high homogeneity in electrical property, which was verified by the spatial uniformity in the resistance mapping of the 36 sensing elements (Fig. S23). All the 36 sensing elements maintained similar electrical properties with an average resistance of 0.81 MΩ (standard deviation is 0.04 MΩ), wherein the maximum and minimum values were 0.88 and 0.76 MΩ, respectively. This favors reliable sensing output over a large area by minimizing interference from serial–parallel resistances.

**Fig. 4.**
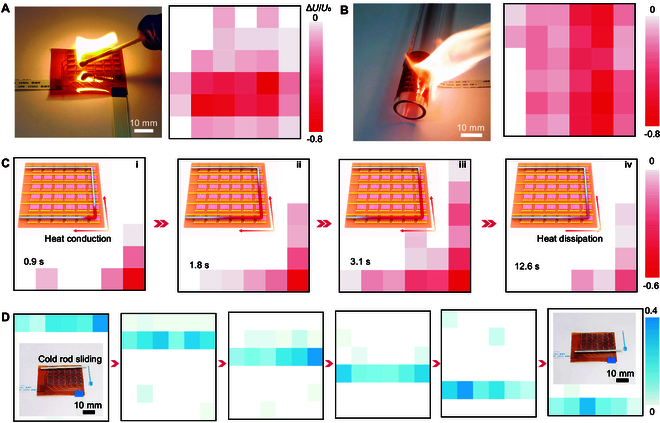
Mo_1−*x*_W*_x_*S_2_ alloy-based temperature sensing array. (A) The left panel shows the photograph of the sensor array being in close contact with a flame from firewood, and the right panel is the corresponding voltage mapping, demonstrating the shape of the flame. The black pixel indicates the closed position of the flame. Relative voltage change is defined as Δ*U*/*U*_0_ = (*U* − *U*_0_)/*U*_0_, where *U*_0_ and *U* are the measured voltages before and after being subjected to external stimuli. The color bars in the following figures are all indicated by the relative voltage changes. (B) The left panel is a photograph of the sensor array under bending deformation. The right panel is the recorded output signals of the bent sensor array being close to a flame from a fire gun. (C) Measured voltage mappings for the sensor array placed on a U-shaped Fe rod with one hot corner (marked by a white dashed circle in the inset) at different times from (i) 0.9 s, (ii) 1.8 s, and (iii) 3.1 s, to (iv) 12.6 s, showing the voltage change induced by the heat conduction and dissipation. Insets are schematics of the sensor array under test. (D) Voltage mappings show the traces of the rod sliding. The white pixels indicate the increased voltages induced by the increase in resistance, which is attributed to the lowered temperature once the sensor array becomes in contact with the cold Fe rod. The voltages roughly recover to the original values after the rod is sliding away. Insets: Photographs of the sensor array when a Fe rod immersed in liquid N_2_ slides on the surface from top to bottom.

We first investigate the capability of the sensor array to distinguish the object shapes by putting a U-shaped iron (Fe) rod on the sensor array. As shown in Fig. S24A and B, there was an increase in the output signal when the Fe rod was immersed in liquid N_2_ for 2 min, while the output signal became negative once the Fe rod was heated using a fire gun. Both of these tests revealed that the shape of the Fe rod was identified as indicated by the color contrast mapping of the same shape. In addition to identifying regular shapes, the sensor array was utilized to verify arbitrary shapes, wherein flames from a fire gun (Movie S4) and firewood (Movie S5) were moving close to the sensor array. The corresponding output signal mappings provided a direct measurement for the temperature distribution of the flames (Fig. 4A and Fig. S24C). Several complex shapes, including star and heart, were also used to validate the spatial mapping of the sensor array. As expected, the mappings matched well with the solid samples (Fig. S24D to F), suggesting a high sensitivity of the sensor array. More importantly, as expected, the sensor array maintained the sensing property even when it was bent with a bending radius of 12.5 mm (left panel in Fig. 4B). As displayed in the right panel in Fig. 4B, the flame shape from a fire gun was identified by the sensor array under bending deformation, further verifying the mechanical flexibility and sensing stability. It should be noted that the ambient humidity and surface humidity of the sensor are expected to have minimal influence on the test results due to its relative insensitivity to relative humidity (less than 3.5% variation in resistance at relative humidity of about 60%; Fig. S25). Beyond identifying object shapes, the sensor array could function as electronic monitors that provide a direct approach to monitoring heat conduction. To this end, a U-shaped Fe rod with one corner being heated to about 620 K was placed on the sensor array (insets in Fig. 4C). It is well known that heat will be conducted to both sides of the Fe rod, resulting in a gradually increased temperature. The corresponding temperature mapping at different times precisely reflected the spatial location information of the conducted heat in the Fe rod (Fig. 4C). Moreover, the variation in the output signal of each sensing pixel could be used for evaluating the temperature of the Fe rod or the conducting speed of heat. Remarkably, the dynamic response of a rod sliding on the surface of the sensor array was also accurately recorded (Fig. 4D), which verified the high sensitivity and fast response of the sensor array. It is worth noting that no capping layers were required in our temperature sensor array to retain sensing performances under harsh conditions, such as low temperature from liquid N_2_ or high temperature from flame burning, demonstrating the potential applications in large-area sensing for extreme temperature monitoring.

## Discussion

Using a printing-and-annealing strategy, we realized a full W composition tuning (*x* = 0 to 1 with steps of 0.2) of Mo_1−*x*_W*_x_*S_2_ alloy patterns on flexible PI substrates, demonstrating tunable thickness (from 5.1 to 46 nm), feature sizes (minimum: 49 μm), and electrical properties (resistance range in about 0.07 to 78.3 MΩ). Resistive-type temperature sensors comprising Mo_1−*x*_W*_x_*S_2_ alloys as sensing layer were capable of achieving an ultrabroad temperature-response range of 20 to 823 K with exceptional sensitivities of 5.02 and −0.88 % K^−1^ at low-temperature and high-temperature range, respectively. The sensor exhibited a rapid response time of about 90 μs, which could detect instantaneous temperature changes induced by contact with liquid N_2_, water droplets, and flames. The sensors also exhibited a high-temperature resolution down to 0.02 K, as well as a reliable response over 100 heating/cooling cycles at various temperature differences from 70 to 470 K. Taking advantage of the proposed approach and the overwhelmingly collective sensing performances, we fabricated a 6 × 6 flexible temperature sensor array, demonstrating the ability to accurately identify arbitrary shapes, recording heat conduction, and monitoring object movement. We believe that the Mo_1−*x*_W*_x_*S_2_ alloy-based temperature sensors can unlock opportunities for future development of temperature sensing under extreme conditions and practical applications in wearable devices.

## Materials and Methods

### Ink formulation

A mixture solution comprising 1,2-propanediol, ethanol, and water was prepared. Then, desired amounts of (NH_4_)_2_MoS_4_ and (NH_4_)_2_WS_4_ (Shanghai Aladdin Bio-Chem Technology Co. Ltd., China) with precisely calculated molar ratios of Mo to W from 1 to 0 at steps of 0.2 were added into the mixture solution to meet the required compositions of the alloys during synthesis, followed by mechanical stirring and ultrasonic treatment for 10 min to complete the dissolution. The amount of (NH_4_)_2_MoS_4_ and (NH_4_)_2_WS_4_ is tailored to prepare alloy precursor inks with concentrations of 2, 5, and 10 mg ml^−1^.

### Inkjet printing and annealing

The prepared inks were deposited onto PI substrates (thickness: 100 μm) through a commercial Fujifilm Dimatix 2850 inkjet printer with a nozzle diameter of 21 μm, a drop volume of 10 pl, and a drop spacing of 25 μm. Before printing, the PI substrates with Au interdigital electrodes were treated with UV/O_3_ for 4 min to enhance the ink spreading. After deposition, the sample was dried at 110 °C for 5 min before being transferred into a quartz furnace for thermal annealing. The printed patterns were then heated at 400 °C for 20 min in a tube furnace, which was filled with a mixture gas of Ar and H_2_ with a flow rate of 60/30 standard cubic centimeter per minute (sccm).

### Fabrication of sensor array

Crossing electrodes were designed to minimize the number of electrical contacts. First, Cr/Au film with a thickness of 5/100 nm was thermally evaporated onto PI substrate, followed by a patterning process for the fabrication of Au interdigital electrodes via a direct laser writing system. The laser speed was 1,000 mm s^−1^, and the laser power was 13%, resulting in the removal of Au film without damage to PI surface. The resultant Au interdigital electrodes had a trace width and spacing of 100 μm. Next, via holes on one branch of Au interdigital electrodes with a diameter of 0.4 mm were drilled using the same laser system with a speed of 100 mm s^−1^ and a laser power of 80%. Following the drilled holes, precursor inks were deposited on the Au interdigital electrodes and annealed to convert to Mo_1−*x*_W*_x_*S_2_ alloys. Then, Ag electrodes and electrical contacts were screen-printed onto the reverse side of the PI substrate to avoid the short of crossing electrodes. Finally, the sensor array was obtained after filling the via holes with Ag inks to connect the Au electrodes and Ag electrodes. In this design, only 12 electrical contacts were required for the sensor array to work properly, instead of 36 contacts for planar electrode design.

### Density functional theory calculation

The ternary Mo_1−*x*_W*_x_*S_2_ alloys were constructed using various W compositions with *x* = 0, 0.14, 0.28, 0.43, 0.57, 0.72, 0.86, and 1, through 2H-MoS_2_ compound with 2 × 2 × 1 supercell. The band structures of Mo_1−*x*_W*_x_*S_2_ alloys were calculated using the following methods and parameters. First-principles calculations were performed using the Vienna Ab-initio simulation package (VASP) with the projector augmented wave method and plane-wave basis (energy cutoff: 500 eV). The generalized gradient approximation (GGA) with the Perdew–Burke–Ernzerhof (PBE)-type exchange-correlation potential was adopted. The Brillouin zone was conducted using the Γ-centered Monkhorst–Pack K-point grids with 5 × 5 × 2 mesh in self-consistent calculations.

### Characterizations and measurements

Raman spectroscopy was employed using WITEC Alpha 200R with a 532-nm laser excitation wavelength. An atomic force microscope (Cypher S) was used to evaluate the thickness. The binding energies of the alloy film were investigated by a Thermo Fisher Scientific ESCALAB 250Xi XPS Microprobe. The XPS results are normalized by S2p_3/2_ peak intensity and calibrated to the C1s peak at 285 eV. The morphology, crystalline structure, and element mapping were evaluated using TEM (FEI Themis Z; accelerating voltage, 300 kV), scanning TEM (STEM), and EDX (JEM 2100F; accelerating voltage, 200 kV). Hall measurements were conducted using a Hall testing system (RH2035). *I*–*V* curves at temperatures from 20 to 80 K and the pulsed heating/cooling tested were conducted by a semiconductor parameter analyzer (FS380 Pro, Platform Design Automation) in a probe station. The resistance of the sensors under low and high temperatures was collected from a digital multisource meter (Keithley 2450, Tektronix). The electrical output of the sensor array was recorded using a homemade multi-channel data acquisition system comprising 16 × 16 channels.

## Data Availability

The data used to support the findings of this study are available within the article and the Supplementary Materials. Raw data are available from the corresponding authors upon reasonable request.

## Supplementary Materials

Supplementary 1 Figs. S1 to S25 Table S1 Movies S1 to S5 References
