# Supplementary material for: Microsecond-Scale Transient Thermal Sensing Enabled by Flexible Mo1−xWxS2 Alloys
Source: Research (Wash D C). 2024 Aug 21;7:0452. doi: 10.34133/research.0452 (PMC11337116; doi:10.34133/research.0452)
Supplement: Supplementary 1 — Figs. S1 to S25 Table S1 Movies S1 to S5 References [file research.0452.f1.zip › research.0422_f1.docx]

**Supplementary Figures**

**
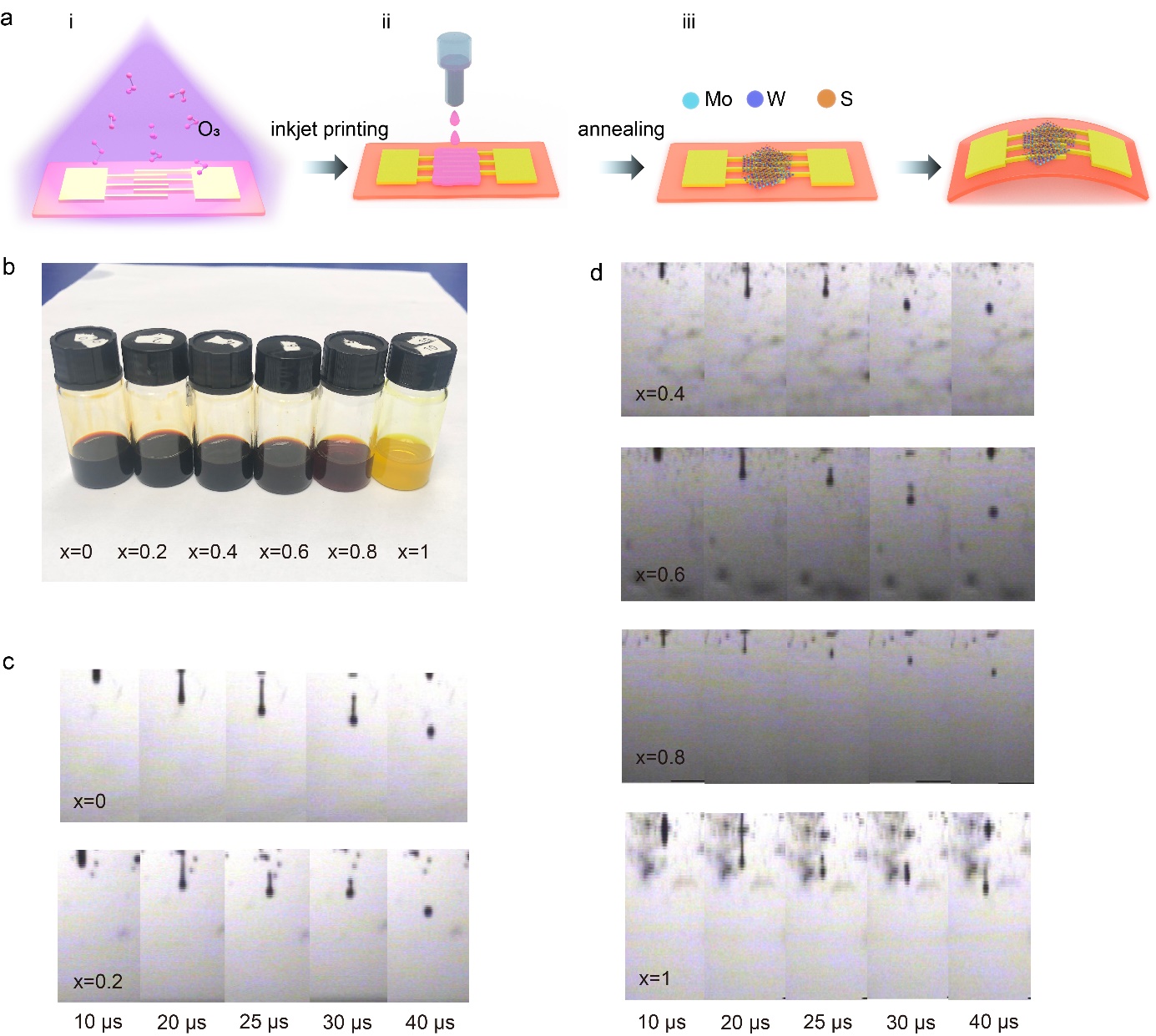
**

**Supplementary Fig. 1. Schematic of the fabrication process, the precursor inks, and jetted droplets in an inkjet printer.** **(a)** Schematic of ink deposition and in situ crystallization to produce Mo1-xWxS2 alloy thin films via an inkjet printing and thermal annealing approach. **(b)** Photograph of the prepared precursor inks with different W compositions from *x*=0 to *x*=1 at steps of 0.2. **(c)** and **(d)** Optical images of the generated droplets from the nozzle of a cartridge for the prepared precursor inks.


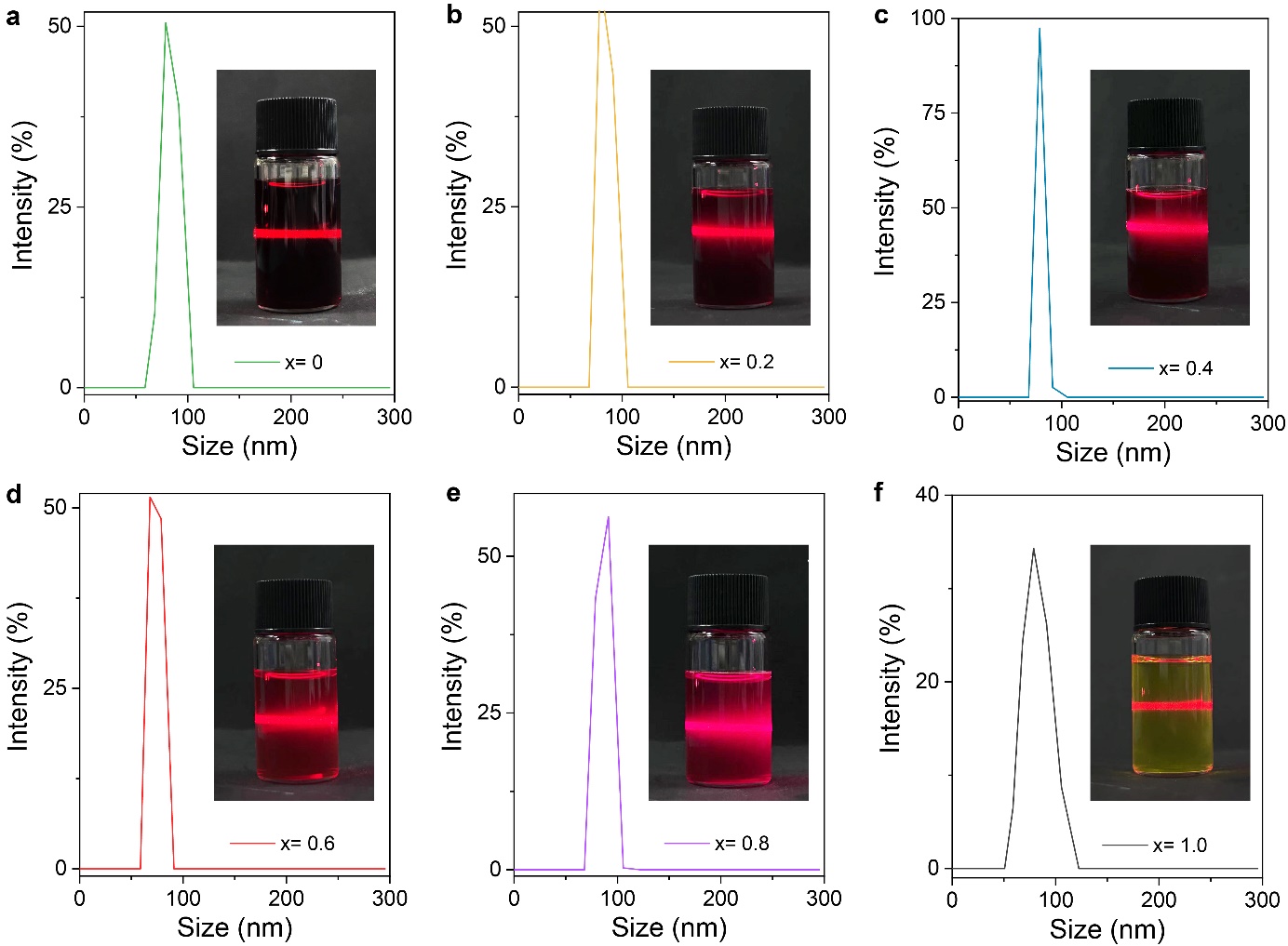


**Supplementary Fig. 2.** Measured size distributions in the precursor inks. Insets show photographs of the inks with a clear Tyndall scattering effect.


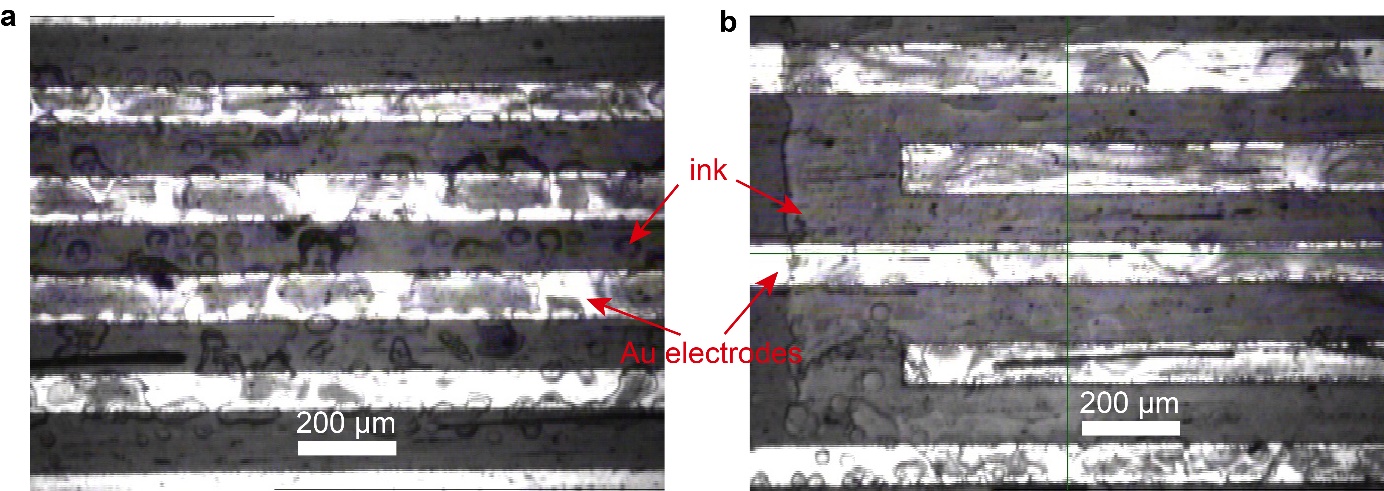


**Supplementary Fig. 3.** Printed patterns on Au interdigital electrodes and PI substrates **(a)** without and **(b)** with UV/O3 treatment for 3 min. Homogeneous and uniform patterns are achieved by depositing precursor ink on the treated substrate.


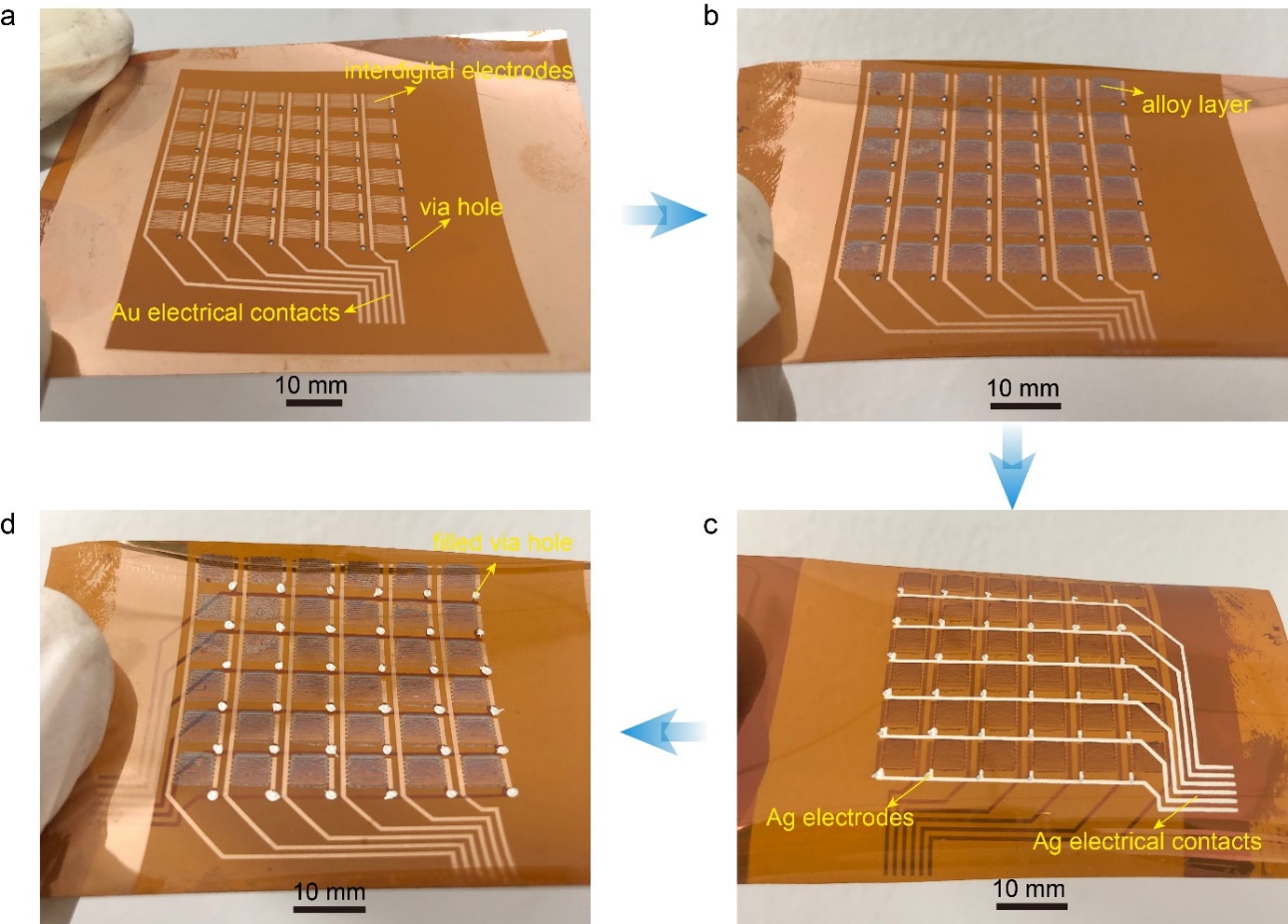


**Supplementary Fig. 4. Photographs of the fabrication process for the sensor array.** **(a)** Thermal deposition of Cr/Au film with a thickness of 5/100 nm, followed by patterning of interdigital electrodes through a laser system equipped with a laser wavelength of 1.06 μm using optimal parameters (laser power: 13%, speed: 1000 mm/s). Via holes with a diameter of 0.4 mm were drilled using the same laser system with a higher laser power (80%) and a low speed (i.e., 200 mm/s). **(b)** Alloy precursor inks were deposited on the interdigital electrodes, followed by thermal decomposition and crystallization to form alloy thin films. **(c)** Ag electrical contacts and electrodes were screen-printed on the reverse side of the PI substrate. The line width was 0.5 mm. The Ag electrical contacts had a line width and line spacing of 0.5 mm for connecting with external instruments for signal recording. **(d)** Via holes were filled with Ag inks by hand, followed by a heating process in an oven at 100 °C for 10 min to complete the fabrication. Using this optimized design, 12 electrical contacts were required for a 6 × 6 sensor array to connect to external parts instead of 72 contacts with a planar structure design.

**
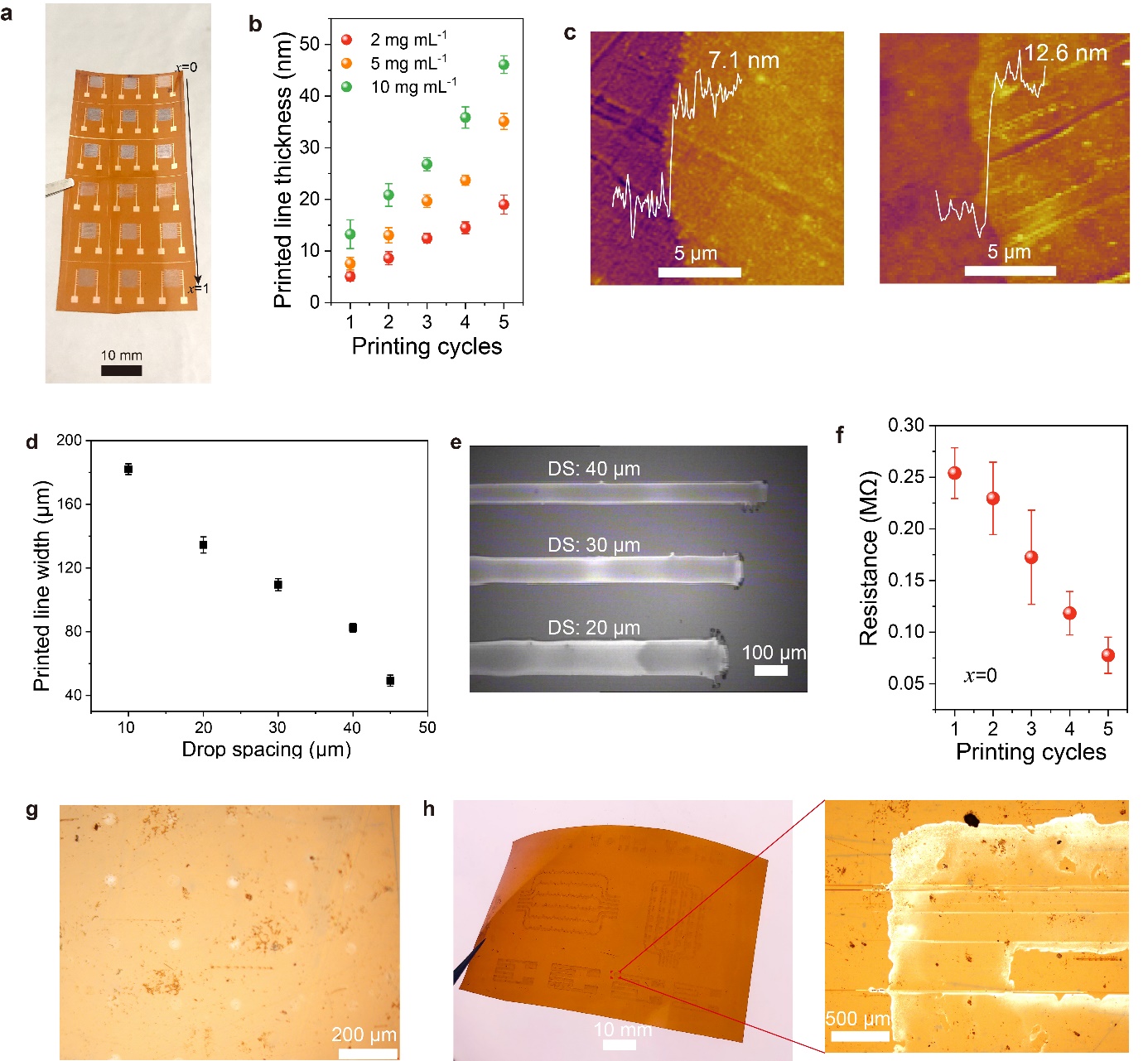
**

**Supplementary Fig. 5. (a)** Photograph of the printed Mo1-xWxS2 alloy thin filmswith different W compositions on a PI substrate. **(b)** Printed line thickness as a function of printing passes of the MoS2 lines with different precursor ink concentrations of ~2, 5, and 10 mg mL-1. **(c)** AFM images of the printed MoS2 lines made with ink concentrations of 5 and 10 mg mL-1 in a single printing pass, respectively. **(d)** Printed line width as a function of drop spacing from 10 to 50 μm. A narrow line width is achieved with larger drop spacing. **(e)** Typical optical images of printed lines with drop spacing of 20, 30, and 40 μm. **(f)** Resistance variation for increasing the printing passes of MoS2 thin films. (**g)** Optical image of the printed dot array with a diameter of about 40 μm. (**h)** Photograph of the printed large-area patterns and optical image of the printed lines.

**
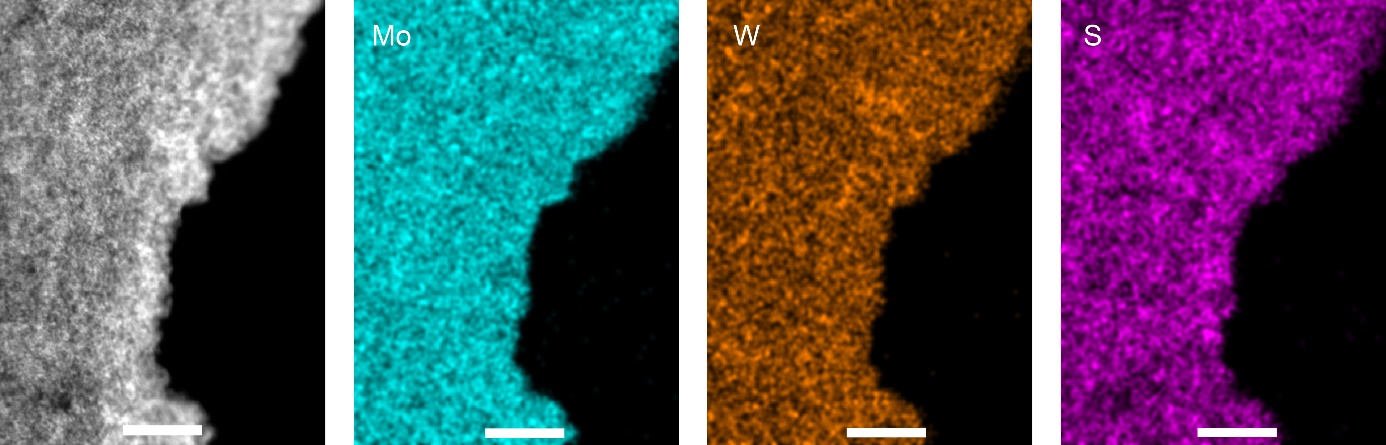
**

**Supplementary Fig. 6.** The EDXS mapping shows the element distributions of Mo, W, and S for the Mo0.6W0.4S2 alloy.


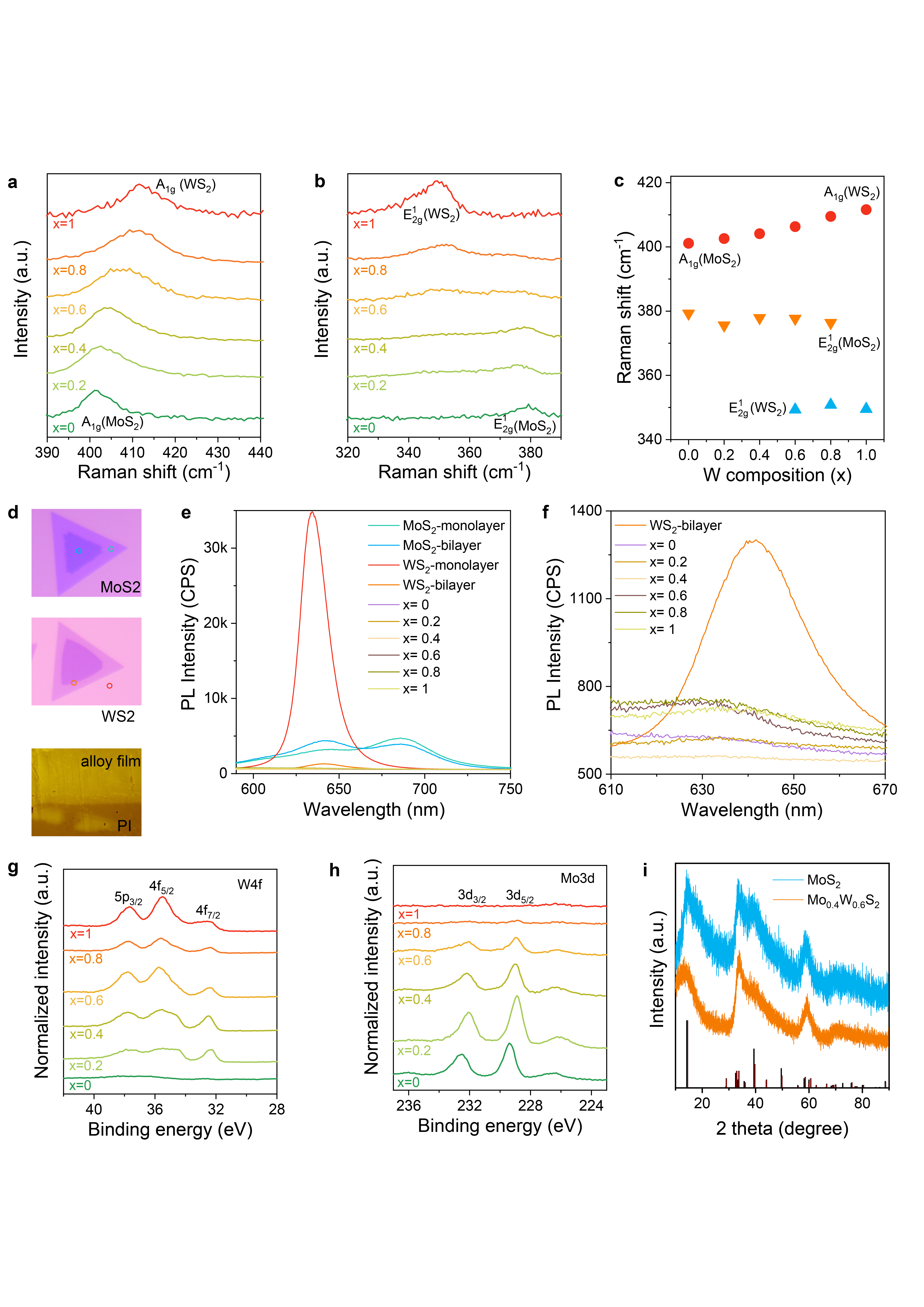


**Supplementary Fig. 7.** (**a-b)** Raman spectra of Mo1-*x*W*x*S2 alloy showing a one-mode and two-mode behavior for *A*1g and modes, respectively. **(c)** The peak variations for the three modes as a function of W composition *x* for the Mo1-*x*W*x*S2 alloy. **(d)** Optical images of MoS2, WS2, and alloy film on PI. **(e)** Layer-dependent PL intensity for monolayer and bilayer MoS2 and WS2, as well as the alloy films with full W composition. **(f)** Enlarged figure in **(e)** showing the extremely weak PL intensity of the alloy films compared to WS2 bilayer. XPS measurements for **(g)** W4f, and **(h)** Mo3d in the Mo1-xWxS2 alloy with different *x*. **(i)** XRD patterns of MoS2 and Mo0.4W0.6S2. The 2-theta positions of MoS2 (black bars, JCPDS 37-1492) and WS2 (red bars, JCPDS 08-0237) are shown for reference.


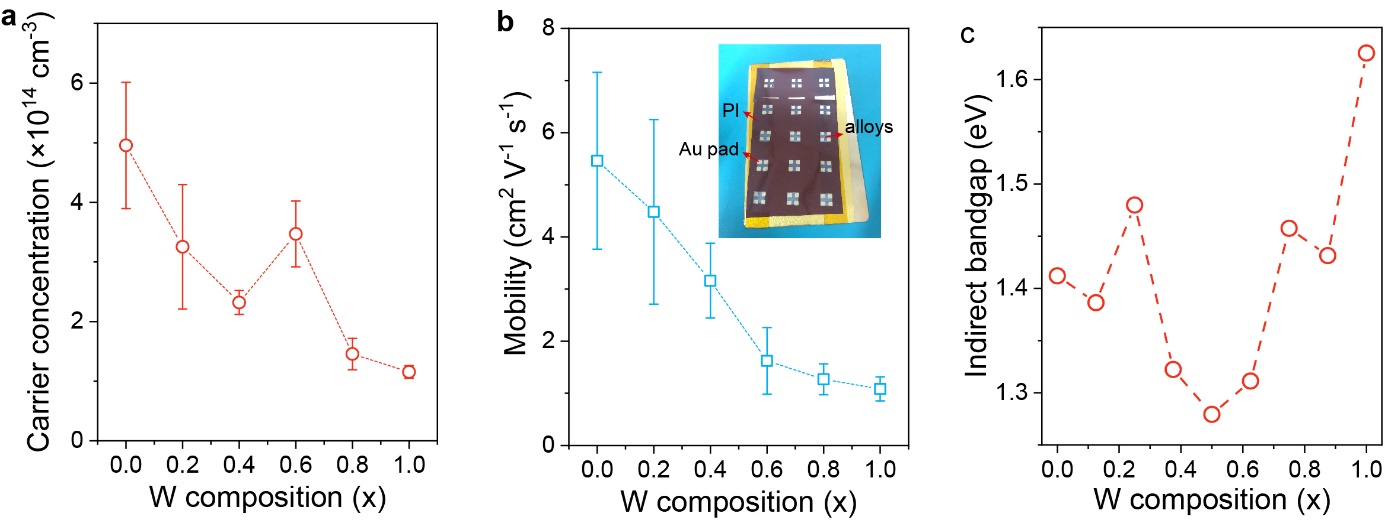


**Supplementary Fig. 8.** Hall measurement to evaluate the **(a)** carrier concentration and **(b)** mobility of the W-doped MoS2 with the full W composition *x*. Inset in **(b)** shows the samples ready for test. **(c)** The calculated bandgaps of Mo1-*x*W*x*S2 alloys with different *x* from 0 to 1 at steps of 0.2. The bandgaps slightly decrease from 1.4 to 1.28 eV at *x*=0 and 0.5, then increase to 1.62 when *x* reaches 1.


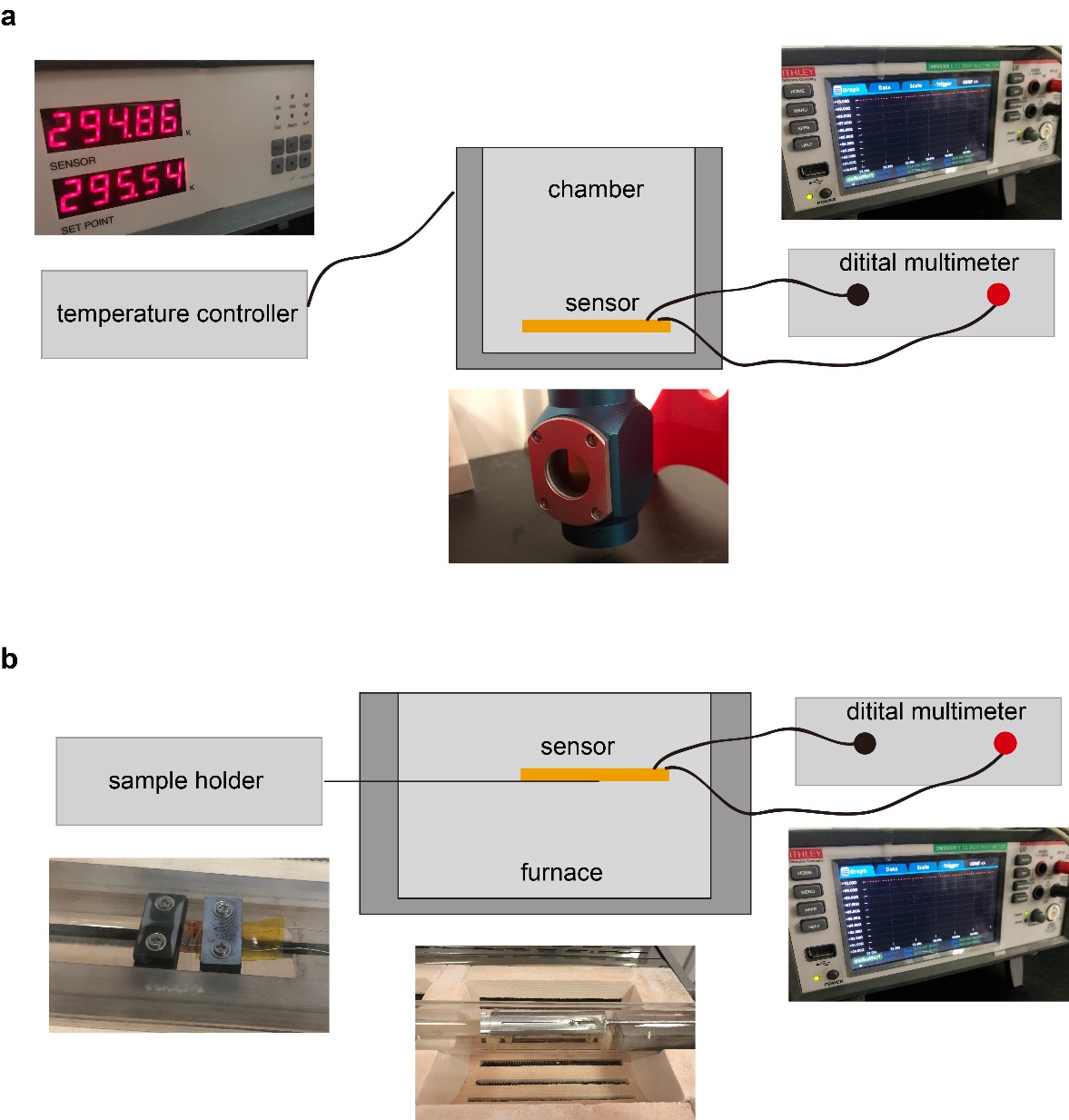


**Supplementary Fig. 9.** Schematic and photographs of the setups used for controlling temperatures **(a)** from 80 to 303 K with a resolution of 0.01 K and **(b)** from 303 to 823 K with a resolution of 1 K. The sensor is placed in a chamber with a precise temperature controller in the low-temperature setup. The electrical output is recorded using a two-source digital multimeter. In the high-temperature setup, a furnace was used to increase the temperature. The sensor was located on a homemade sample holder and the resistance change versus temperature was recorded by a two-source source meter.


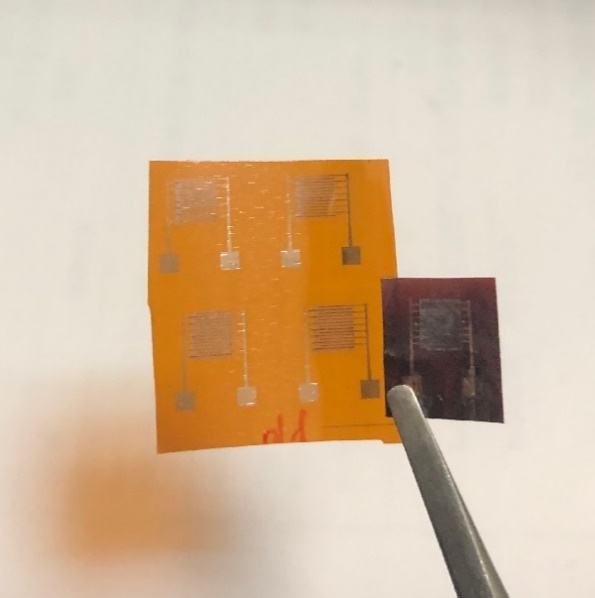


**Supplementary Fig. 10.** Photograph of the flexible sensors before (left) and after (right) thermal treatment at 860 K for one hour, showing the color change of the PI film.


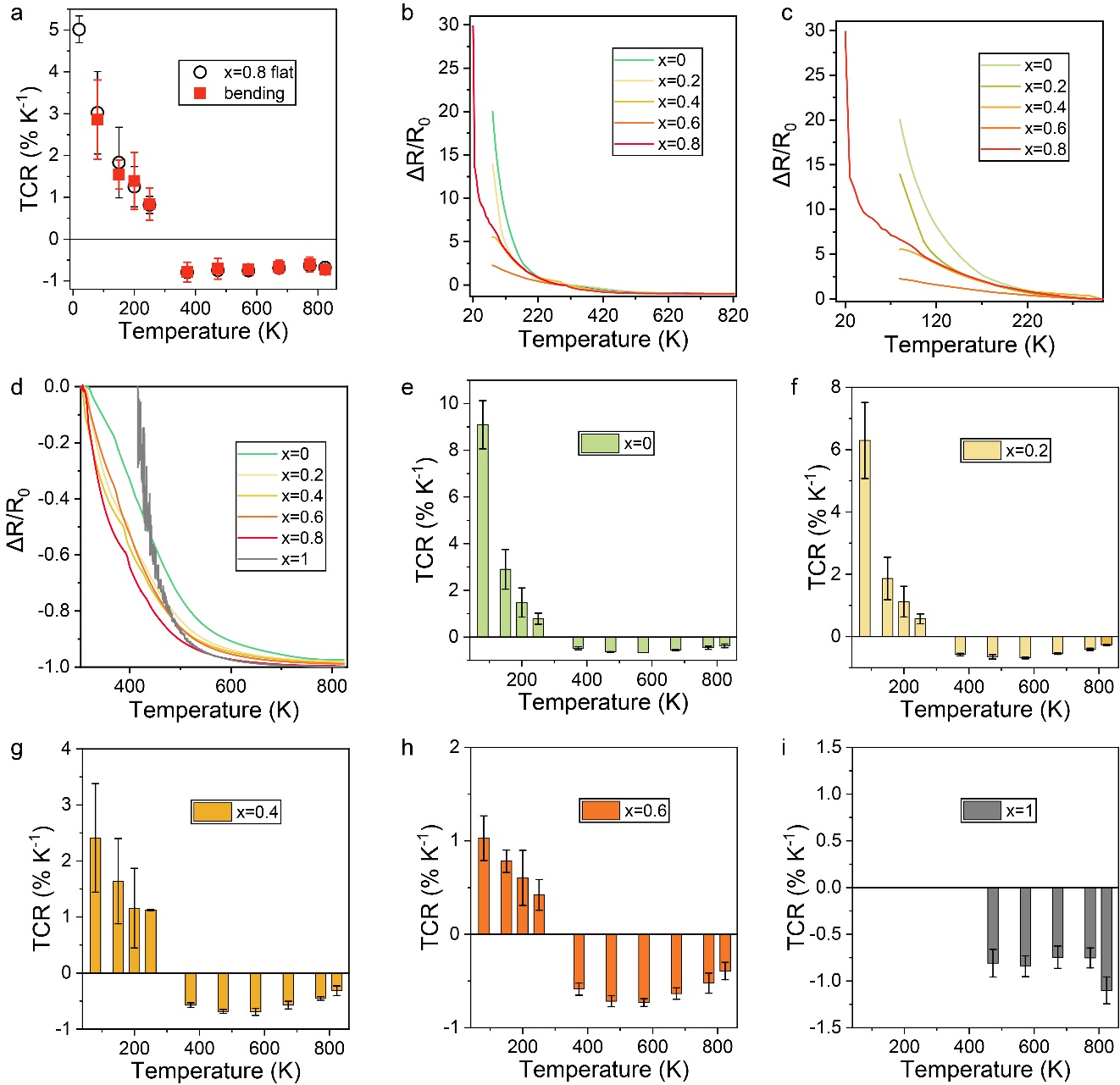


**Supplementary Fig. 11. The relative resistance change and calculated temperature coefficient of resistance (TCR) for the Mo1-*x*W*x*S2 alloys-based sensors with different W composition.** **(a)** Calculated TCR of temperature sensor using Mo0.8W0.2S2 alloy as sensing element at flat and bending deformations. The relative resistance change of the Mo1-*x*W*x*S2 alloys-based temperature sensors over a wide temperature range **(b)** from 20 to 823 K, **(c)** 20-303 K and **(d)** 303-823 K. Since WS2 has no conductivity below about 420 K, the relative resistance change of WS2-based sensor is not measured below 420 K. The calculated TCR of the Mo1-*x*W*x*S2 alloys-based temperature sensors with **(e)** *x*=0, **(f)** *x*=0.2, **(g)** *x*=0.4, **(h)** *x*=0.6, and **(i)** *x*=1.


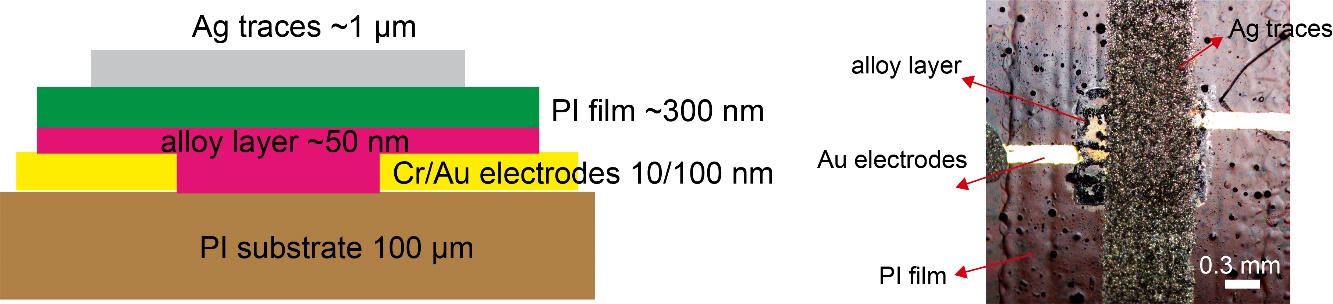


**Supplementary Fig. 12.** Schematic (left panel) and optical image (right panel) of the designed structure for the measurement of response time. A PI film with a thickness of about 300 nm was printed on the surface of the alloy layer as an insulator to avoid the electrical short of Au interdigital electrodes after printing the heater. An Ag line was printed on top of the PI film, which generated heat after being applied to a pulse voltage. The generated heat from the heater was then spreaded to the alloy layer through the PI film, resulting in a change in the resistance of the sensor.


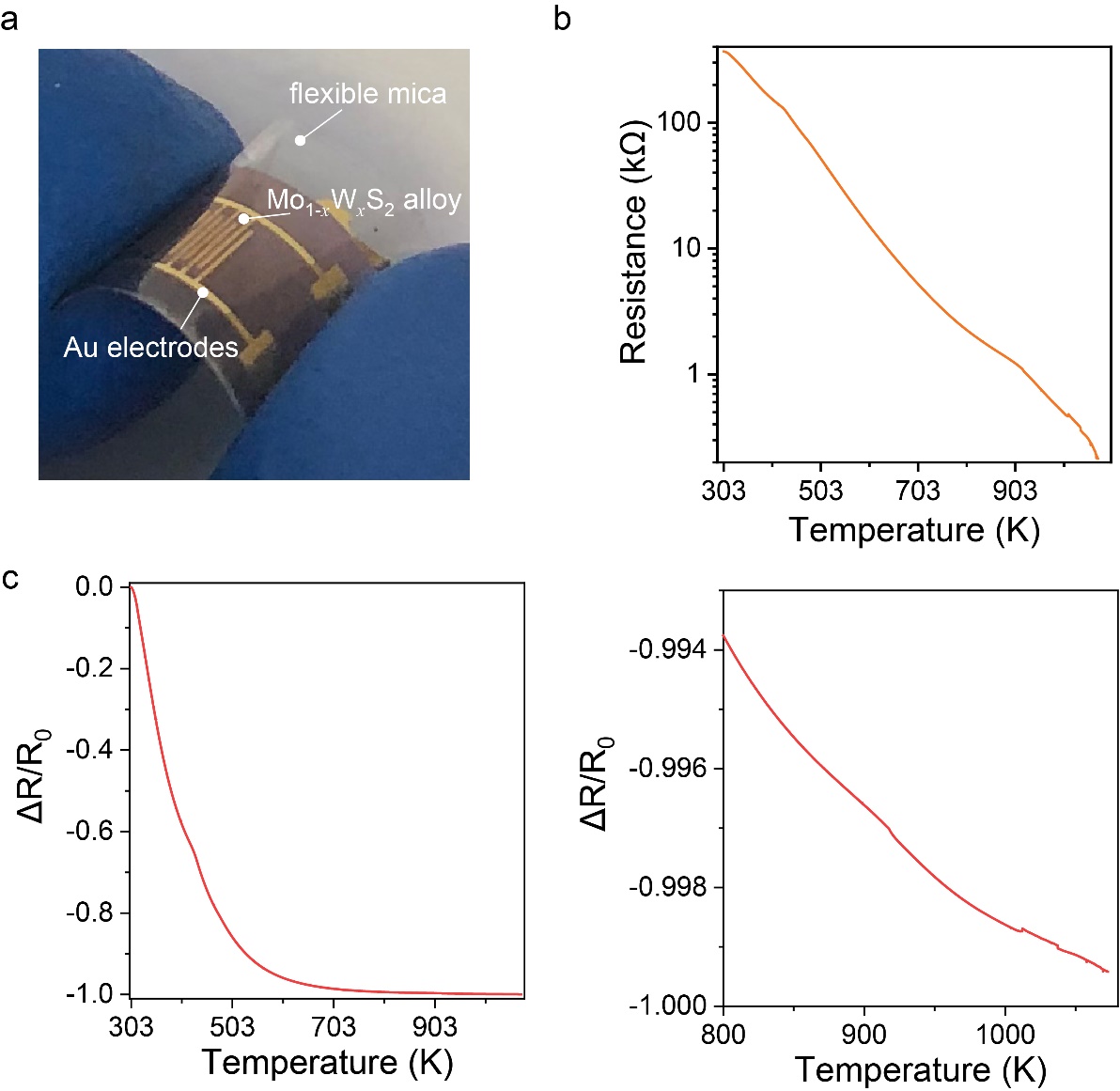


**Supplementary Fig. 13.** Photograph and electrical properties of flexible temperature sensor. **(a)** Photograph showing Mo0.8W0.2S2 alloy layer on flexible mica substrate. **(b)** Measured resistance as a function of temperature from 303 to 1073 K. **(c)** Relative resistance change in resistance of the sensor at temperatures from 303 to 1073 K. **(d)** Relative resistance change in resistance of the sensor at temperatures from 800 to 1073 K.


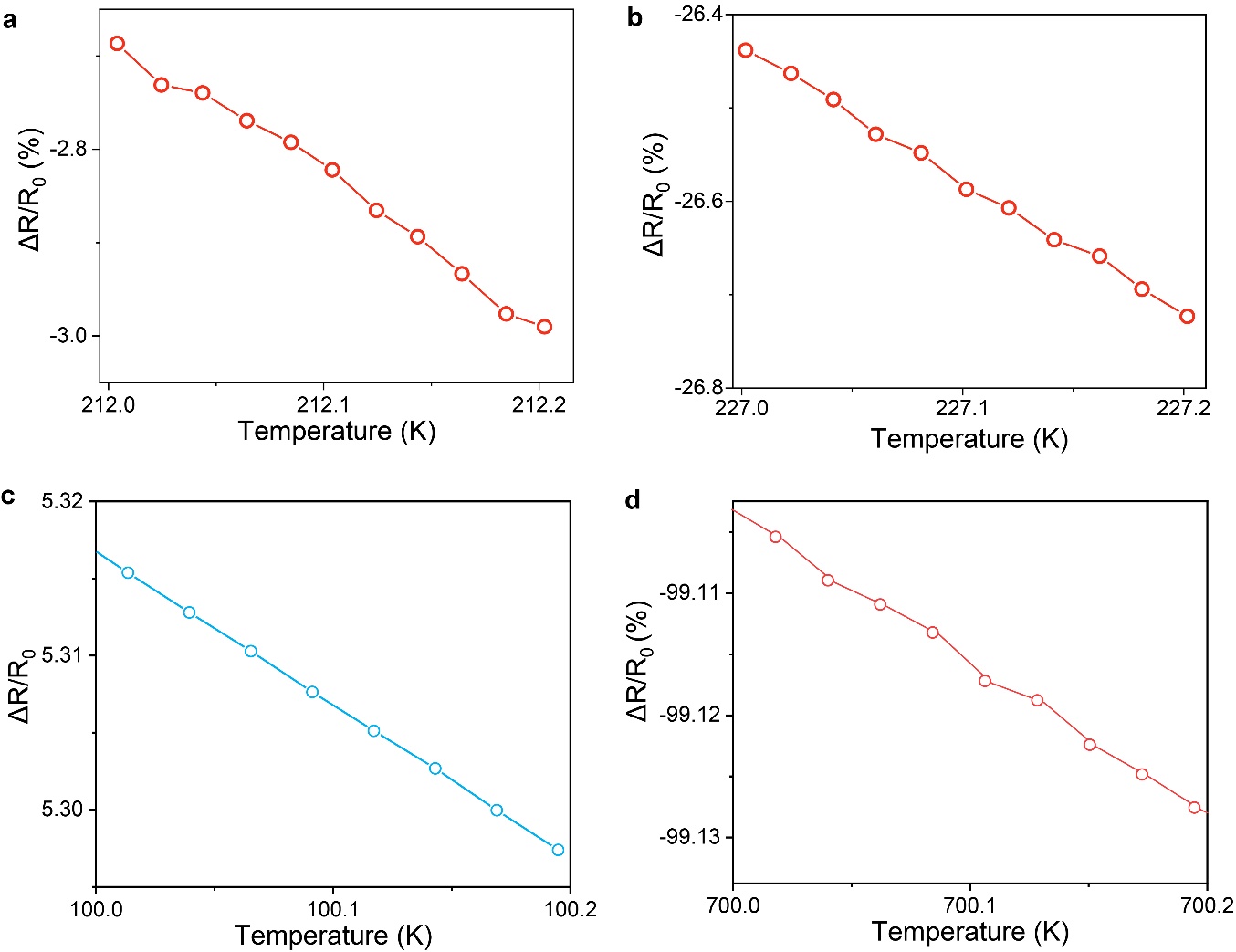


**Supplementary Fig. 14.** The relative change in resistance of the sensor at temperatures of **(a)** 212 to 212.2 K, **(b)** 227 to 227.2 K with steps of 0.02 K, **(c)** 100 to 100.2 K, and **(d)** 700 to 700.2 K with steps of about 0.025 K. The results show the successful detection of subtle temperature variation.


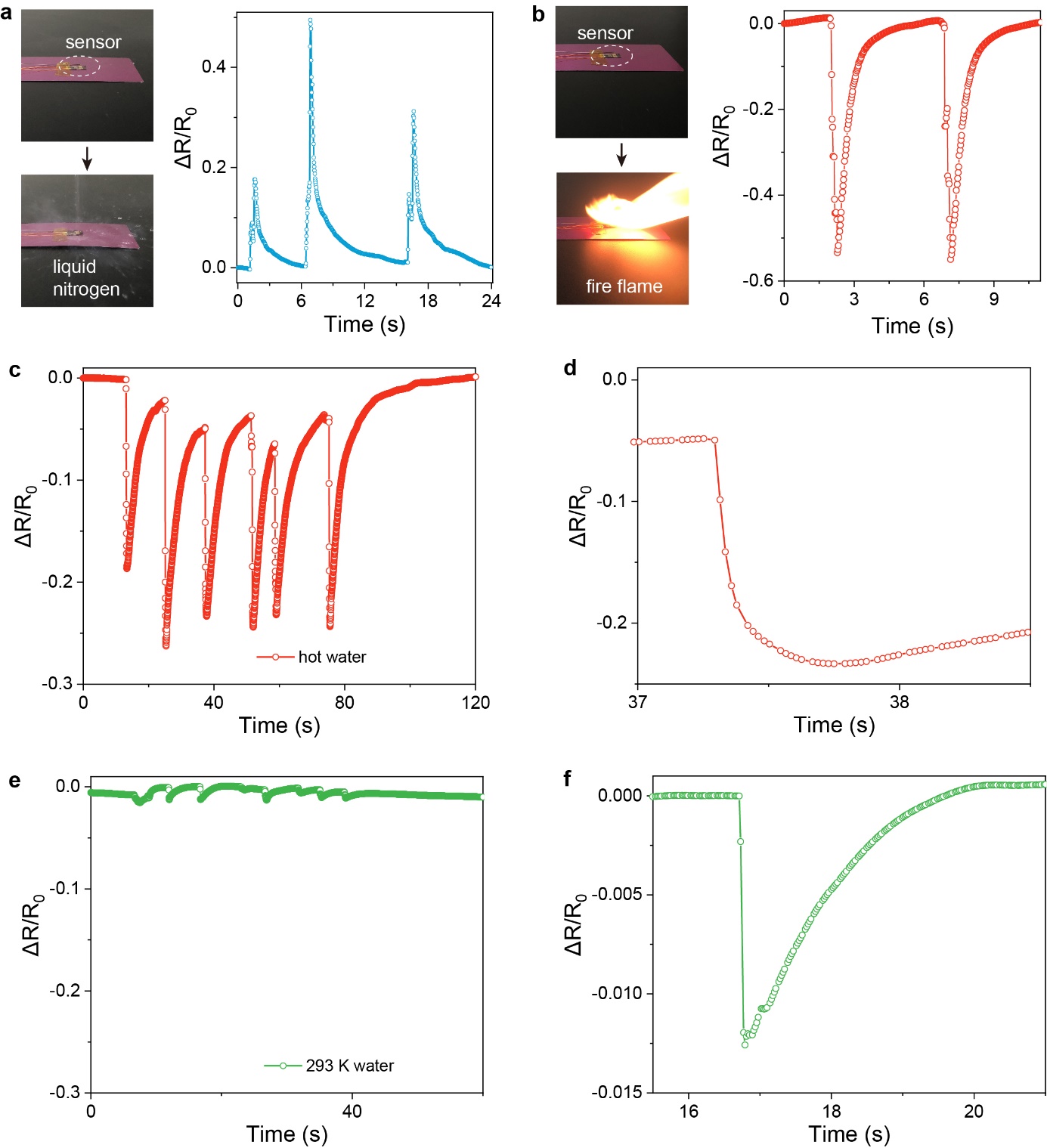


**Supplementary Fig. 15.** Reliable and reversible response of the sensor to the contact with (**a)** liquid N2, and **(b)** fire flame. The left panels show the photographs of the sensors under test. **(c)** Relative change of resistance versus time for the sensor to come into contact with hot water droplets. An instantaneous decrease in resistance was observed once the droplets touched the surface of the sensors. Compared to liquid nitrogen, the recovery was relatively slow, which was attributed to the slow evaporation of water. **(d)** The enlarged curve from 37 to 38.5 s in a, showing that the temperature saturation for the sensor was within 0.1 s. In other words, a fast response time of about 0.1 s was required to reach the temperature balance between hot water and the sensor. **(e)** The relative change of resistance when the sensor was in close contact with room-temperature water droplets. **(f)** The enlarged sensing outpt for the sensor in 16 to 20 s, showing a fast saturation time of 60 ms.


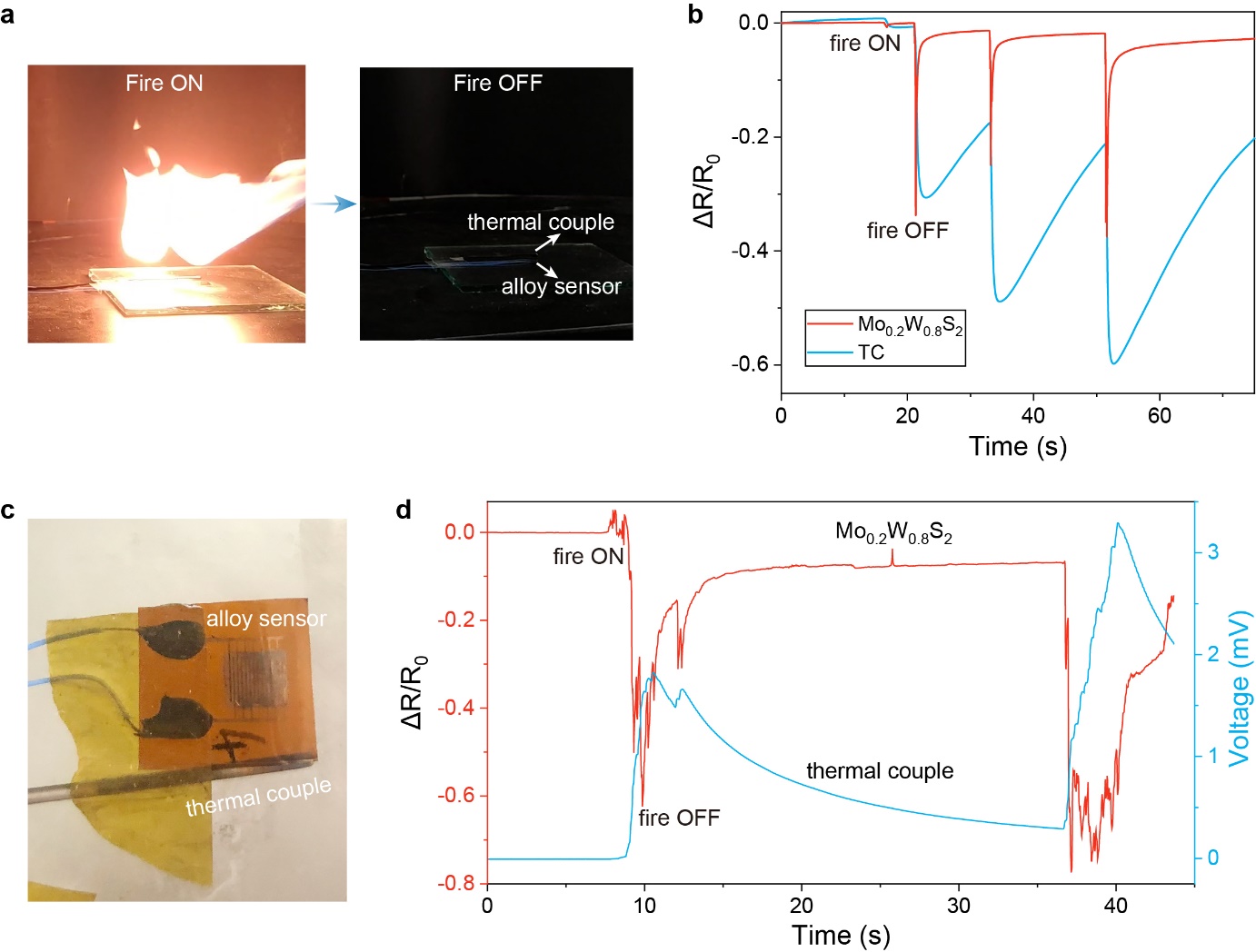


**Supplementary Fig. 16. Comparison of response and resolution for the proposed sensor and commercial thermocouples.** **(a)** Photographs of the sensor and thermocouple during and after burning by a fire flame. **(b)** The relative change in resistance of the two sensors subjected to the same fire flame over three cycles. The proposed alloy-based sensor exhibited a faster response and recovery. **(c)** Photograph of the sensor and thermocouple under test. **(d)** The relative change in resistance of the two sensors when a fire flame was applied to the sensors. The proposed alloy-based sensor clearly identified the fluctuation of the fire flame, which was not observed by the thermocouple.


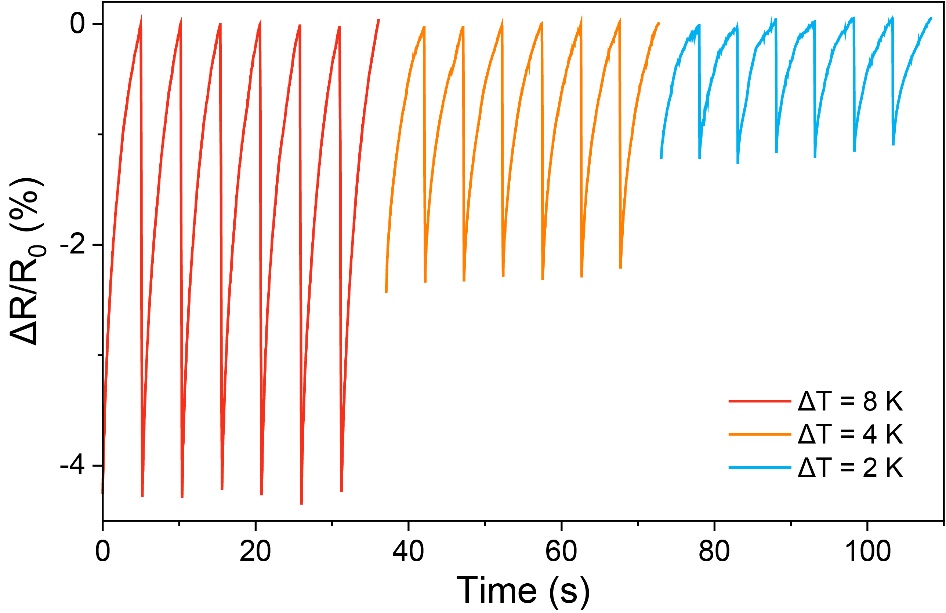


**Supplementary Fig. 17.** The relative change of resistance when the sensor is applied to identical temperature gradients of 2, 4, and 8 K.


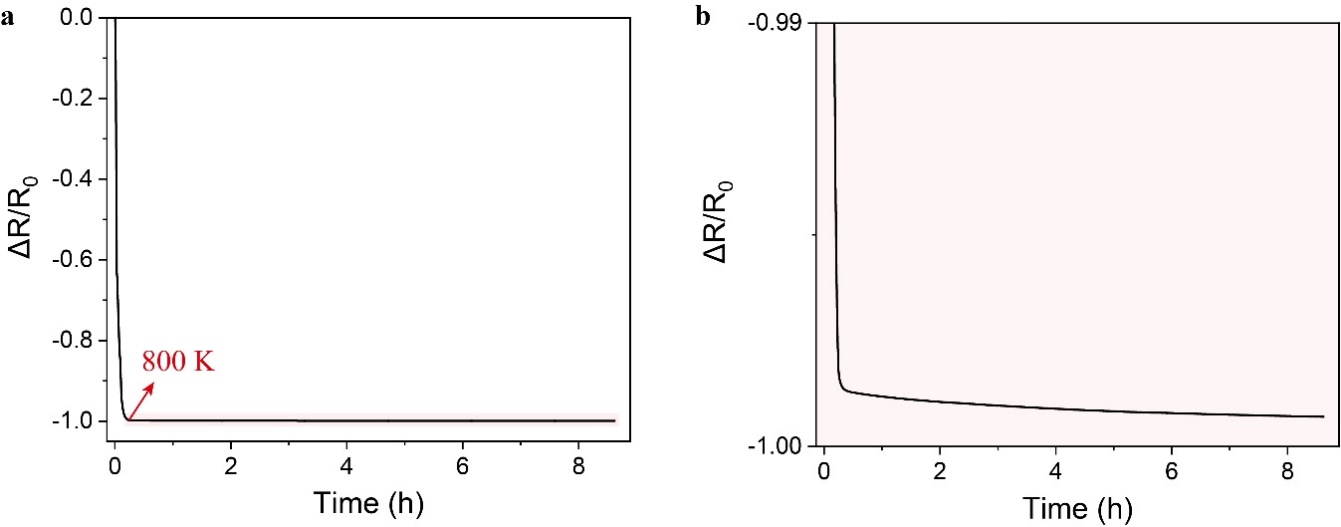


**Supplementary Fig. 18.** Long-term stability of the alloy sensor. **(a)** the relative resistance change of the alloy-based temperature sensor at a high temperature of 800 K. **(b)** The magnified figure of the red box in **a** shows the stable output signal for a long time over 8 h.


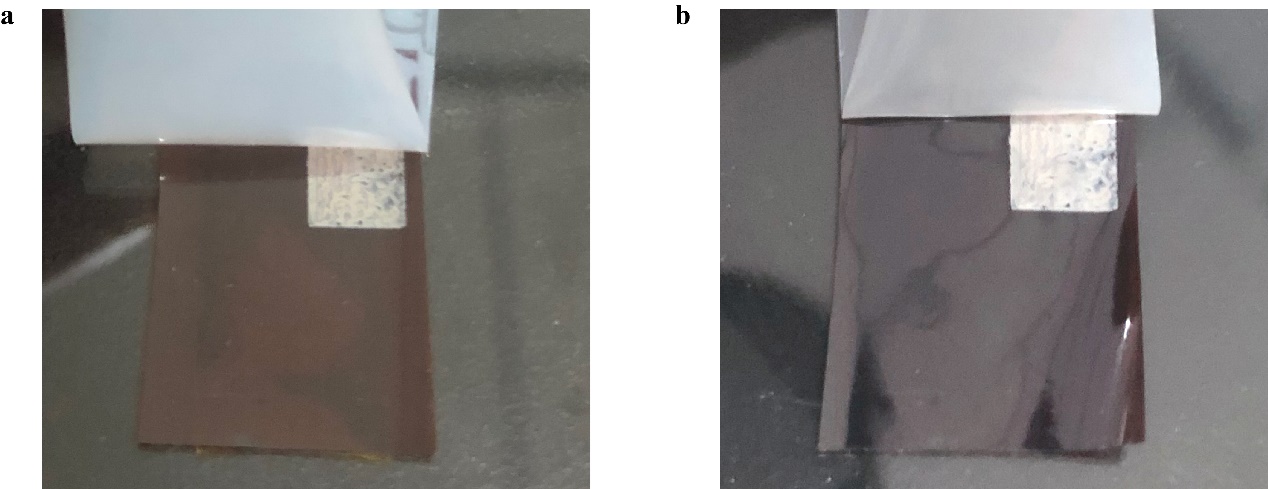


**Supplementary Fig. 19.** Photographs of the alloy films with a tape peeling test **(a)** before and **(b)** after the thermal experiment at 823 K for one hour. It shows that there is neither delamination nor damage of the alloy layer, demonstrating strong adhesion of the layer with PI film.


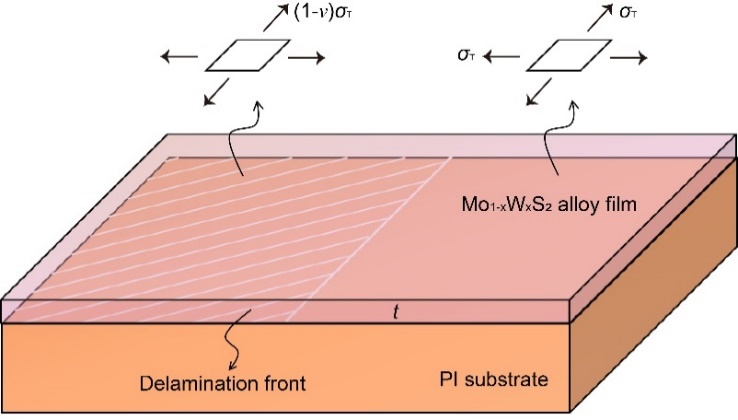


**Supplementary Fig. 20.** Schematic illustration of the thermal stress within the alloy film on PI substrate.


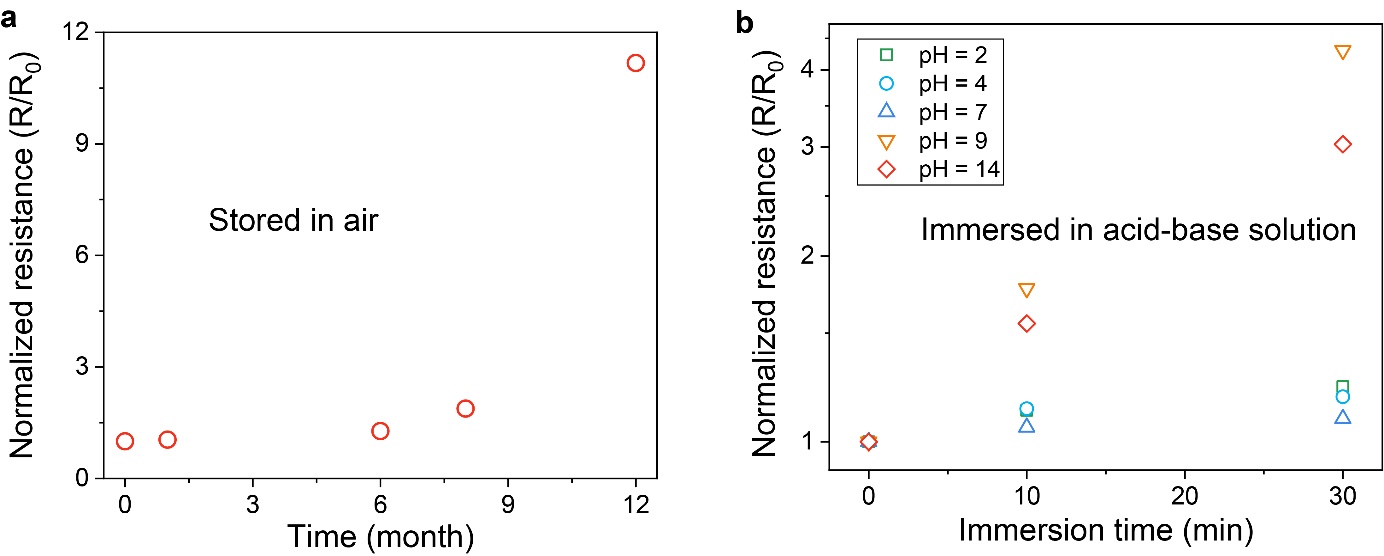


**Supplementary Fig. 21. a,** Normalized resistance of the sensor after being stored in air at a temperature of 25 ℃ and relative humidity of about 60% for 6, 8, and 12 months. **b,** Normalized resistance of the sensor after being immersed in acid and alkaline solutions with pH values from 2 to 14.


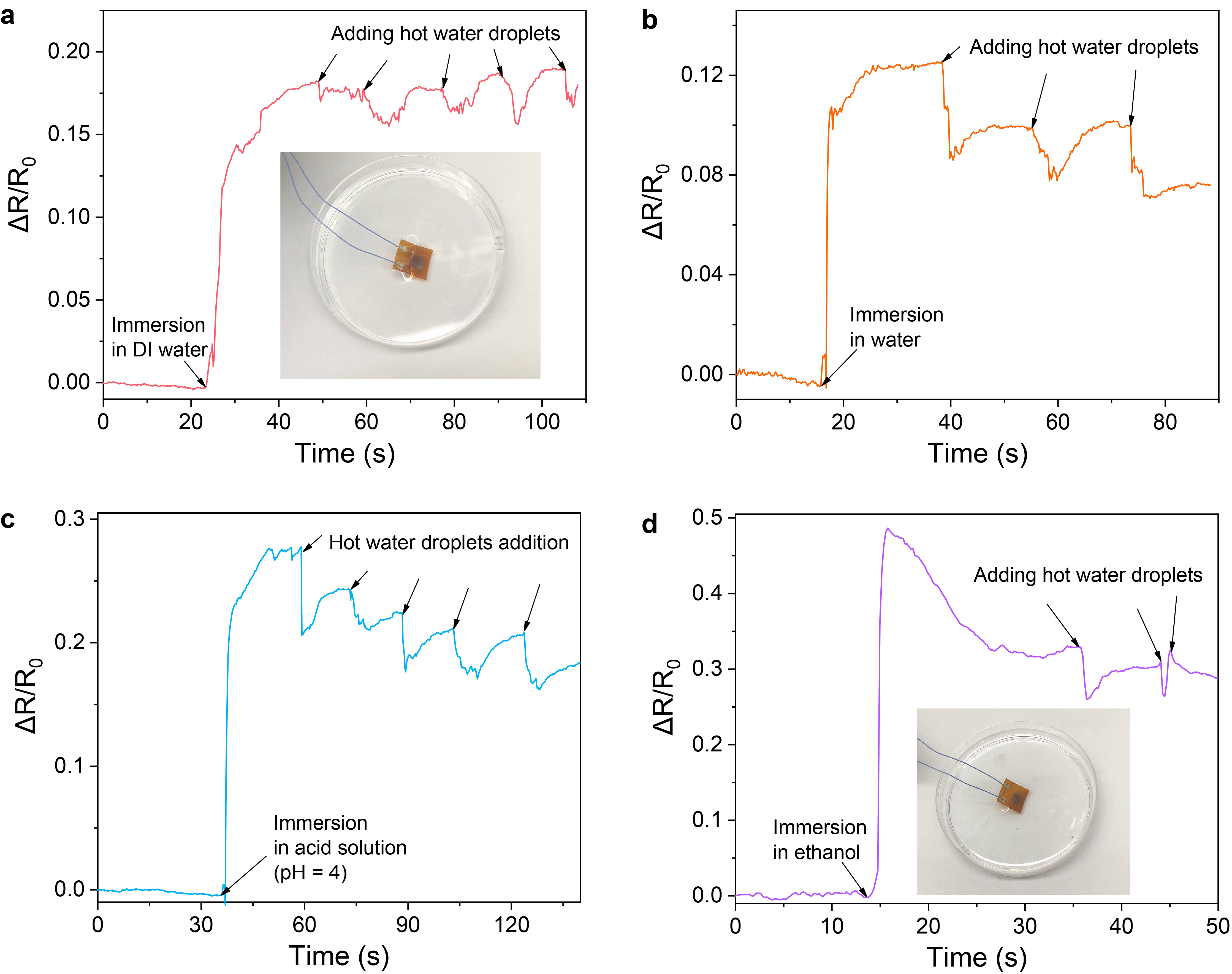


**Supplementary Fig. 22.** The relative change of resistance when the sensor was immersed into **a,** deionized (DI) water, **b,** drinking water, **c,** acid solution (pH = 4), and **d,** ethanol. A few droplets of hot water were also added to the solutions to investigate the response of the sensor. It shows that there is decreased resistance upon the addition of hot water droplets, indicating the increased temperature. These results validate the proper working of the sensor in various solutions.


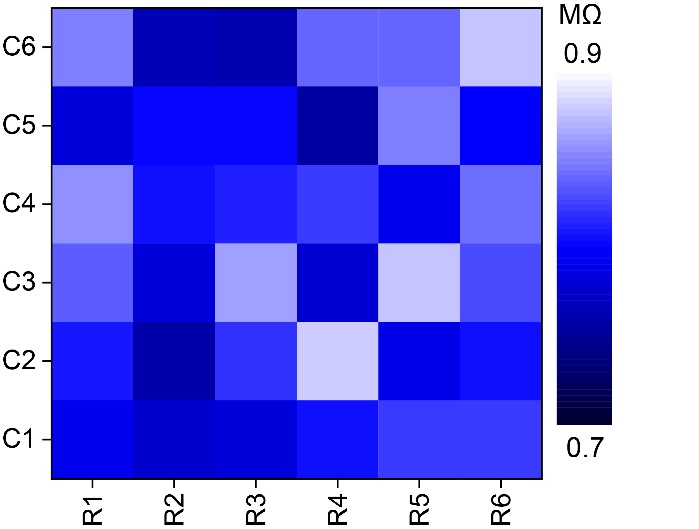


**Supplementary Fig. 23.** Resistance distribution of the sensing elements for a 6 × 6 sensor array, exhibiting a relatively uniform resistance, ranging from 0.7 to 0.9 MΩ.


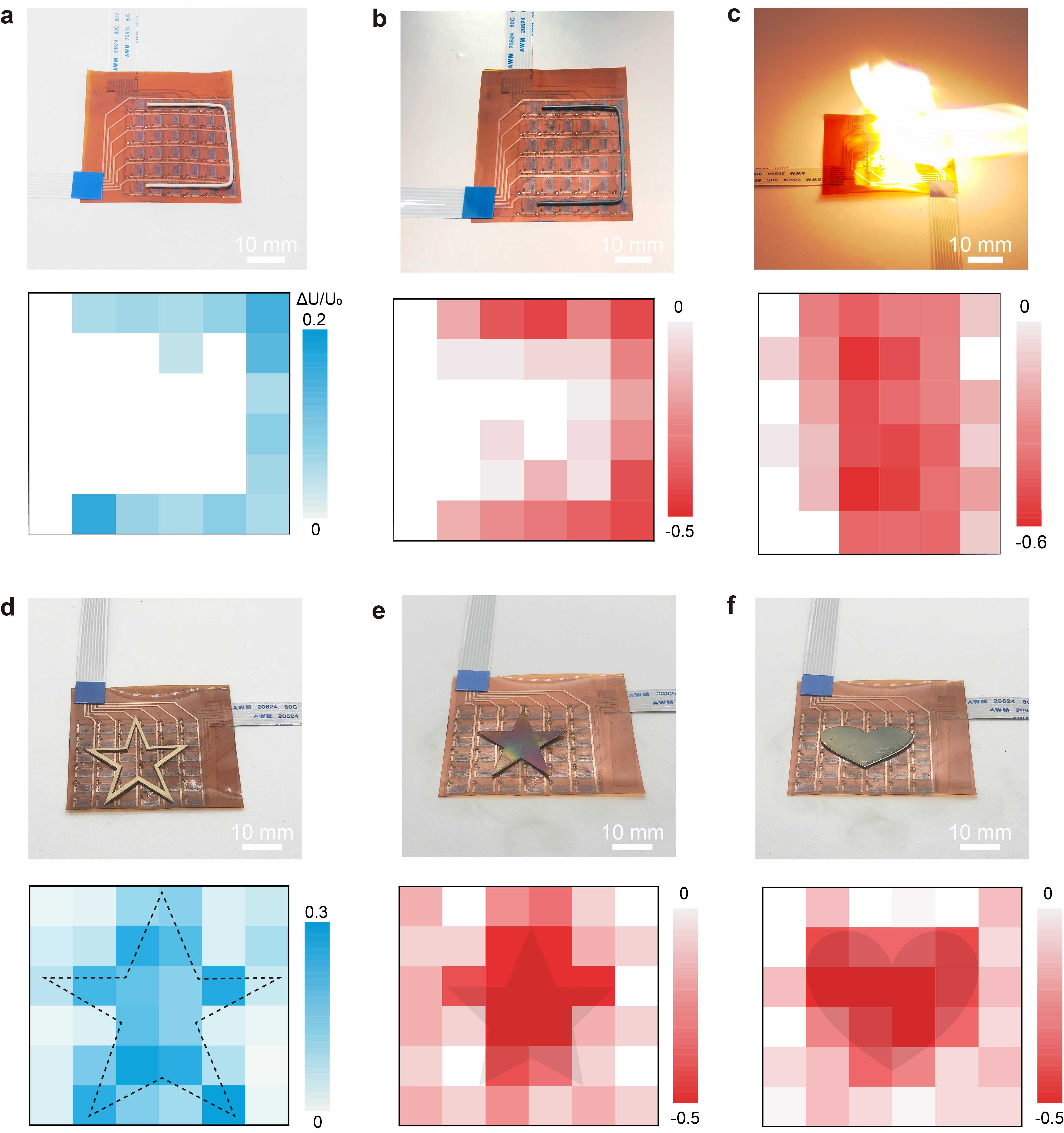


**Supplementary Fig. 24.** Photographs and the corresponding output signal mappings for the sensor array placed on a **(a)** U-shaped cold Fe rod and **(b)** U-shaped hot Fe rod. **(c)** Photograph and the corresponding output signal mappings for the sensor array subjected to a fire flame from a fire gun. Photographs and the corresponding output signal mappings for the sensor array placed on a **(d)** cold star, **(e)** hot star, and **(f)** hot heart shapes. All the signal mappings reflected the object shapes regardless of the regular or arbitrary shapes.


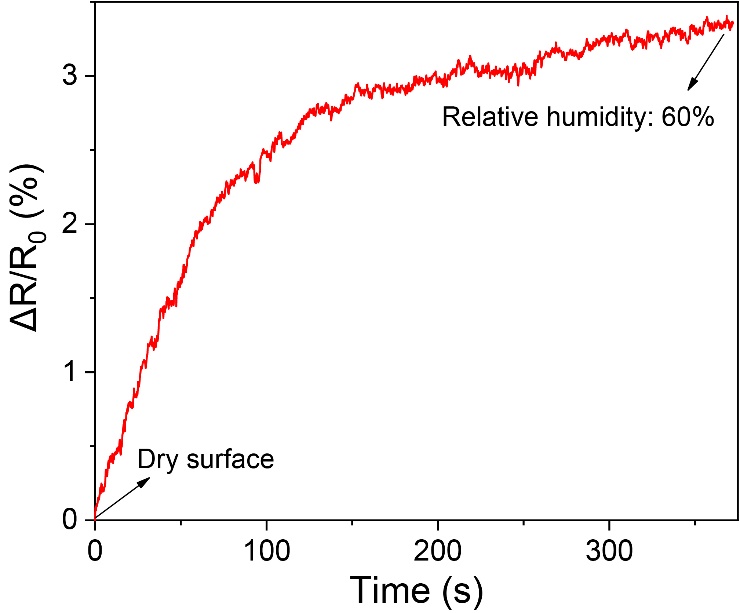


**Supplementary Fig. 25.** The relative resistance variation of the sensor with dry surface and wet surface (relative humidity of about 60%).

**Table S1. Comparison of TCR, response time, resolution, and work temperature range for different materials in the literature and the proposed sensors.**

| Material type | materials | TCR  (% K-1) | Response time (s) | Sensing resolution | Sensing range (°C) | Ref. |
| --- | --- | --- | --- | --- | --- | --- |
| inorganic semiconductor | Ag2S | -4.7 | 0.11 | 0.05 | 25-80 | [1] |
|  | MoS2 | 1-2 | 0.000036 | 0.1 | 27-120 | [2] |
|  | MoS2 | 0.53 | - | - | 100-300 K | [3] |
|  | MoTe2 | 0.63 | - | - | 100-300 K | [3] |
|  | SnSe2 | -0.43 | 0.3 | - | 28-40 | [4] |
|  | Ni | 0.4 | - | - | 25-70 | [5] |
|  | NiO | -9.2 | 0.05 | - | 25-70 | [5] |
|  | Ni/NiO | 0.38 | - | - | 25-100 | [6] |
|  | black phosphorus | 0.173 | 0.5 | - | 25-50 | [7] |
| metals | Au | 0.03 | 0.013 | 0.02 | 22-50 | [8] |
|  | Cr/Au | 0.25 | 0.0042 | - | 25-50 | [8] |
|  | Ag NPs | 2-5 | - | 0.4 | 20-100 | [9] |
|  | Ag | 0.37 | 2.5 |  | 25-100 | [10] |
|  | Pt | 0.145 | 0.08 | - | 0-400 | [11] |
|  | Au@AgNW–PEG–PUa | 1.6 | 100 | 0.5 | 25-75 | [12] |
| organic semiconductor | PANI nanofiber | -1.6 | 19.5 | 2.7 | 40-110 | [13] |
|  | PANI/Graphene | 1.6 | 0.7 | 0.3 | 25-40 | [14] |
|  | PEDOT:PSS | -0.77 | 1.5 |  | 25-50 | [15] |
|  | PEDOT:PSS/PANI | -0.83 | 0.2 | 0.1 | 32-42 | [16] |
|  | PEDOT:PSS/CNT | 0.25 | 1 | - | 20-80 | [17] |
|  | PEDOT:PSS | 0.09 | 0.09 | 1.22 | 25-90 | [18] |
| carbon nanomaterials | rGO-PU | 1.34 | 10 | 0.2 | 30-80 | [19] |
|  | CNT-GO | 6 |  |  | 10-40 | [20] |
|  | rGO | 0.74 | - | - | 25-150 | [21] |
|  | rGO | 2.04 | 22 | 0.2 | 26-101 | [22] |
|  | rGO | 0.801 | 52 | 0.1 | 30-100 | [23] |
|  | rGO fiber | 0.636 | 7 | - | 30-80 | [24] |
|  | rGO hydrogel | 2.04 |  | 0.2 | 26-101 | [25] |
|  | CNT | 0.07 | 20 | 2 | 4-420 K | [26] |
|  | rubber-CNT | 1.6 | 50 | - | 30-100 | [27] |
|  | carbon nanofiber | 1.52 | 1.2 | 1 | 30-55 | [28] |
|  | carbon nanofiber | -2.44 | 0.73 | 1 | 25-50 | [29] |
|  | Graphene | 0.42 | - | - | 25-50 | [30] |
|  | Graphene | 0.17 | - | - | 30-45 | [31] |
| gels | hydrogel | 1.43 | 20 | 1 | 20-70 | [32] |
|  | gel | 8.42 | 0.61 | - | 30-90 | [33] |
| **This work** | **Mo1-*x*W*x*S2** | **5.02/ -0.88** | **0.00003/**  **0.00009** | **0.02** | **20-823 K** |  |

aPEG and PU indicate poly-ethylene glycol and polyurethane, respectively.

**Other Supplementary Material for this manuscript includes the following:**

Movie S1 (.mp4 format). Demonstration of the response of the sensor to liquid nitrogen.

Movie S2 (.mp4 format). Demonstration of the response for the sensor to fire flame.

Movie S3 (.mp4 format). Demonstration of the response for the sensor and a commercial thermocouple to fire flame.

Movie S4 (.mp4 format). Demonstration of the 6 × 6 sensor array to identify the shape of fire flame.

Movie S5 (.mp4 format). Demonstration of the 6 × 6 sensor array to identify the shape of a firewood.

**Supplementary References**

1. X. F. Zhao, S. Q. Yang, X. H. Wen, Q. W. Huang, P. F. Qiu, T. R. Wei, H. Zhang, J. C. Wang, D. W. Zhang, X. Shi, A Fully Flexible Intelligent Thermal Touch Panel Based on Intrinsically Plastic Ag2S Semiconductor. *Adv. Mater.* 2022; 34: 2107479.

2. A. Daus, M. Jaikissoon, A. I. Khan, A. Kumar, R. W. Grady, K. C. Saraswat, E. Pop, Fast-Response Flexible Temperature Sensors with Atomically Thin Molybdenum Disulfide. *Nano Lett.* 2022; 22: 6135-6140.

3. A. I. Khan, P. Khakbaz, K. A. Brenner, K. K. H. Smithe, M. J. Mleczko, D. Esseni, E. Pop, Large temperature coefficient of resistance in atomically thin two-dimensional semiconductors. *Appl. Phys. Lett.* 2020; 116: 203105.

4. S. Veeralingam, S. Badhulika, 2D-SnSe2 nanoflakes on paper with 1D-NiO gate insulator based MISFET as multifunctional NIR photo switch and flexible temperature sensor. *Mater. Sci. Semicond. Process.* 2020; 105: 104738.

5. J. Shin, B. Jeong, J. Kim, V. B. Nam, Y. Yoon, J. Jung, S. Hong, H. Lee, H. Eom, J. Yeo, Sensitive wearable temperature sensor with seamless monolithic integration. *Adv. Mater.* 2020; 32: 1905527.

6. A. B. Appiagyei, J. Banua, J. I. Han, Flexible and patterned-free Ni/NiO-based temperature device on cylindrical PET fabricated by RF magnetron sputtering: Bending and washing endurance tests. *J. Ind. Eng. Chem.* 2021; 100: 372-382.

7. A. Chhetry, S. Sharma, S. C. Barman, H. Yoon, S. Ko, C. Park, S. Yoon, H. Kim, J. Y. Park, Black Phosphorus@Laser-Engraved Graphene Heterostructure-Based Temperature–Strain Hybridized Sensor for Electronic-Skin Applications. *Adv. Funct. Mater.* 2021; 31: 2007661.

8. R. C. Webb, A. P. Bonifas, A. Behnaz, Y. Zhang, K. J. Yu, H. Cheng, M. Shi, Z. Bian, Z. Liu, Y.-S. Kim et al., Ultrathin conformal devices for precise and continuous thermal characterization of human skin. *Nat. Mater.* 2013; 12: 938-944.

9. X. Ren, K. Pei, B. Peng, Z. Zhang, Z. Wang, X. Wang, P. K. Chan, A low‐operating‐power and flexible active‐matrix organic‐transistor temperature‐sensor array. *Adv. Mater.* 2016; 28: 4832-4838.

10. J. M. Nassar, M. D. Cordero, A. T. Kutbee, M. A. Karimi, G. A. T. Sevilla, A. M. Hussain, A. Shamim, M. M. Hussain, Paper Skin Multisensory Platform for Simultaneous Environmental Monitoring. *Adv. Mater. Technol.* 2016; 1: 1600004.

11. Y. Moser, M. A. Gijs, Miniaturized flexible temperature sensor. *J. Microelectromech. Syst.* 2007; 16: 1349-1354.

12. A. Kumar, M. O. Shaikh, R. K. R. Kumar, K. Dutt, C.-T. Pan, C.-H. Chuang, Highly sensitive, flexible and biocompatible temperature sensor utilizing ultra-long Au@AgNW-based polymeric nanocomposites. *Nanoscale.* 2022; 14: 1742-1754.

13. G. Ge, Y. Lu, X. Qu, W. Zhao, Y. Ren, W. Wang, Q. Wang, W. Huang, X. Dong, Muscle-inspired self-healing hydrogels for strain and temperature sensor. *ACS Nano.* 2020; 14: 218-228.

14. H. Liu, K. Sun, X.-L. Guo, Z.-L. Liu, Y.-H. Wang, Y. Yang, D. Yu, Y.-T. Li, T.-L. Ren, An ultrahigh linear sensitive temperature sensor based on PANI:Graphene and PDMS hybrid with negative temperature compensation. *ACS Nano.* 2022; 10.1021/acsnano.2c10342.

15. Y.-F. Wang, T. Sekine, Y. Takeda, K. Yokosawa, H. Matsui, D. Kumaki, T. Shiba, T. Nishikawa, S. Tokito, Fully printed PEDOT: PSS-based temperature sensor with high humidity stability for wireless healthcare monitoring. *Sci. Rep.* 2020; 10: 1-8.

16. J. Song, Y. Wei, M. Xu, J. Gao, L. Luo, H. Wu, X. Li, Y. Li, X. Wang, Highly Sensitive Flexible Temperature Sensor Made Using PEDOT:PSS/PANI. *ACS Appl. Polym. Mater.* 2022; 4: 766-772.

17. S. Harada, K. Kanao, Y. Yamamoto, T. Arie, S. Akita, K. Takei, Fully printed flexible fingerprint-like three-axis tactile and slip force and temperature sensors for artificial skin. *ACS Nano.* 2014; 8: 12851-12857.

18. T. Bücher, R. Huber, C. Eschenbaum, A. Mertens, U. Lemmer, H. Amrouch, Printed temperature sensor array for high-resolution thermal mapping. *Sci. Rep.* 2022; 12: 14231.

19. T. Q. Trung, S. Ramasundaram, B. U. Hwang, N. E. Lee, An all‐elastomeric transparent and stretchable temperature sensor for body‐attachable wearable electronics. *Adv. Mater.* 2016; 28: 502-509.

20. B. Zhao, V. S. Sivasankar, A. Dasgupta, S. Das, Ultrathin and Ultrasensitive Printed Carbon Nanotube-Based Temperature Sensors Capable of Repeated Uses on Surfaces of Widely Varying Curvatures and Wettabilities. *ACS Appl. Mater. Interfaces.* 2021; 13: 10257-10270.

21. P. Sahatiya, S. K. Puttapati, V. V. S. S. Srikanth, S. Badhulika, Graphene-based wearable temperature sensor and infrared photodetector on a flexible polyimide substrate. *Flexible Printed Electron.* 2016; 1: 025006.

22. J. Wu, W. Huang, Y. Liang, Z. Wu, B. Zhong, Z. Zhou, J. Ye, K. Tao, Y. Zhou, X. Xie, Self-calibrated, sensitive, and flexible temperature sensor based on 3D chemically modified graphene hydrogel. *Adv. Electron. Mater.* 2021; 7: 2001084.

23. P. Sehrawat, Abid, S. S. Islam, P. Mishra, Reduced graphene oxide based temperature sensor: Extraordinary performance governed by lattice dynamics assisted carrier transport. *Sens. Actuators, B.* 2018; 258: 424-435.

24. T. Q. Trung, H. S. Le, T. M. L. Dang, S. Ju, S. Y. Park, N.-E. Lee, Freestanding, Fiber-Based, Wearable Temperature Sensor with Tunable Thermal Index for Healthcare Monitoring. *Adv. Healthcare Mater.* 2018; 7: 1800074.

25. J. Wu, W. Huang, Y. Liang, Z. Wu, B. Zhong, Z. Zhou, J. Ye, K. Tao, Y. Zhou, X. Xie, Self‐Calibrated, Sensitive, and Flexible Temperature Sensor Based on 3D Chemically Modified Graphene Hydrogel. *Adv. Electron. Mater.* 2021; 7: 2001084.

26. A. Di Bartolomeo, M. Sarno, F. Giubileo, C. Altavilla, L. Iemmo, S. Piano, F. Bobba, M. Longobardi, A. Scarfato, D. Sannino, Multiwalled carbon nanotube films as small-sized temperature sensors. *J. Appl. Phys.* 2009; 105: 064518.

27. M. Lin, Z. Zheng, L. Yang, M. Luo, L. Fu, B. Lin, C. Xu, A high-performance, sensitive, wearable multifunctional sensor based on rubber/CNT for human motion and skin temperature detection. *Adv. Mater.* 2022; 34: 2107309.

28. J.-H. Lee, H. Chen, E. Kim, H. Zhang, K. Wu, H. Zhang, X. Shen, Q. Zheng, J. Yang, S. Jeon et al., Flexible temperature sensors made of aligned electrospun carbon nanofiber films with outstanding sensitivity and selectivity towards temperature. *Mater. Horiz.* 2021; 8: 1488-1498.

29. J. H. Lee, E. Kim, H. Zhang, H. Chen, H. Venkatesan, K. Y. Chan, J. Yang, X. Shen, J. Yang, S. Jeon, Rational design of all resistive multifunctional sensors with stimulus discriminability. *Adv. Funct. Mater.* 2022; 32: 2107570.

30. S. Kabiri Ameri, R. Ho, H. Jang, L. Tao, Y. Wang, L. Wang, D. M. Schnyer, D. Akinwande, N. Lu, Graphene electronic tattoo sensors. *ACS Nano.* 2017; 11: 7634-7641.

31. G. Rajan, J. J. Morgan, C. Murphy, E. Torres Alonso, J. Wade, A. K. Ott, S. Russo, H. Alves, M. F. Craciun, A. I. S. Neves, Low Operating Voltage Carbon–Graphene Hybrid E-textile for Temperature Sensing. *ACS Appl. Mater. Interfaces.* 2020; 12: 29861-29867.

32. S. Hao, R. Dai, Q. Fu, Y. Wang, X. Zhang, H. li, X. Liu, J. Yang, A Robust and Adhesive Hydrogel Enables Interfacial Coupling for Continuous Temperature Monitoring. *Adv. Funct. Mater.* 2023: 2302840.

33. P. Yao, Q. Bao, Y. Yao, M. Xiao, Z. Xu, J. Yang, W. Liu, Environmentally Stable, Robust, Adhesive, and Conductive Supramolecular Deep Eutectic Gels as Ultrasensitive Flexible Temperature Sensor. *Adv. Mater.* 2023: 2300114.
